# Gold nanoparticles in the diagnosis and treatment of ovarian and cervical cancers: a comprehensive understanding

**DOI:** 10.3389/fonc.2025.1664340

**Published:** 2025-09-12

**Authors:** Senhui Zhang, Tong Li, Deshuo Jiang, Hengmei Shi, Huyang Hou, Ziyi Fu, Xiaoyan Shi

**Affiliations:** ^1^ Department of Obstetrics and Gynecology, Women’s Hospital of Nanjing Medical University, Nanjing Women and Children’s Healthcare Hospital, Nanjing, China; ^2^ Department of Breast Cancer Center, Affiliated Cancer Hospital of Nanjing Medical University, Jiangsu Cancer Hospital & Jiangsu Institute of Cancer Research, Nanjing, China

**Keywords:** gynecological malignant tumors, cervical cancer, ovarian cancer, nanotechnology, gold nanoparticles, diagnosis, treatment

## Abstract

Cervical and ovarian cancers pose a significant global threat to women’s health. Despite substantial medical advances in recent decades, gynecological malignancies remain a leading cause of female mortality, constrained by factors such as multidrug resistance, treatment toxicity, asymptomatic presentation in early stages, and genetic heterogeneity. Gold nanoparticles (AuNPs), leveraging their exceptional biocompatibility and multifunctional capabilities, demonstrate considerable potential across diverse fields including bioimaging, liquid biopsy, photothermal therapy, and targeted chemotherapy, thereby advancing precision oncology. Accordingly, this review synthesizes and analyzes the emerging applications of AuNPs in gynecological tumors over the past five years. Moving beyond superficial descriptions of functional features often limited in previous reviews, it places greater emphasis on elucidating the intrinsic relationships and mechanisms between functions from the perspective of their physicochemical properties. It further highlights the critical importance of AuNPs for constructing integrated diagnostic and therapeutic platforms. Simultaneously, this review provides a balanced examination of the challenges hindering the clinical translation of AuNPs and offers insights and perspectives on addressing these issues. It is anticipated that AuNPs may evolve into highly effective diagnostic and therapeutic strategies in the future.

## Graphical Abstract

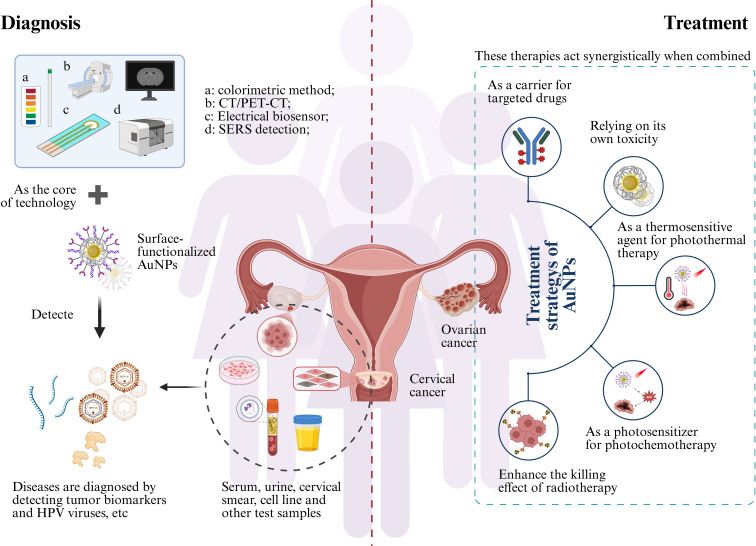

## 1 Introduction

Cervical and ovarian cancers represent the most lethal gynecological malignancies, each posing distinct clinical challenges. HPV infection constitutes the primary etiological factor for most cervical carcinomas, thus establishing HPV testing as critical for early screening (1). Organized screening and HPV vaccination provide key prevention strategies, especially in developing nations (2). The protracted asymptomatic latency spanning decades from cervical intraepithelial neoplasia to invasive carcinoma creates significant fertility preservation challenges for reproductive-aged patients, given that radical hysterectomy remain primary therapeutic options (3, 4). Ovarian cancer demonstrates the highest aggressiveness among gynecological malignancies, with its characteristically asymptomatic early-stage presentation resulting in fewer than 50% of patients surviving beyond five years post-diagnosis (5, 6). Molecular heterogeneity, intrinsic chemoresistance, and rapid metastatic dissemination collectively contribute to its elevated mortality (7).

Conventional strategies lack early precision and fail to prevent multidrug resistance, necessitating advanced approaches (8, 9). With advancements in oncology, nanotechnology has emerged as a promising frontier (10, 11). Therapeutically, multifunctional nanoparticle-based drug delivery platforms enable cancer cell-specific targeting while sparing healthy tissues, thereby reducing systemic drug exposure, minimizing toxicity, and delaying resistance emergence (12–14). Diagnostically, nanoparticles enhance tumor biomarker detection sensitivity, facilitating earlier clinical intervention (15, 16).

Among diverse nanomaterials, gold nanoparticles (AuNPs) stand out due to exceptional biocompatibility and their defining optical property (17). AuNPs are synthesized through established methods including the Turkevich citrate reduction, biological synthesis using plant/microbial extracts, and physical approaches like laser ablation, enabling precise control over size, morphology, and surface functionalization for biomedical applications (18). Localized Surface Plasmon Resonance (LSPR) arises from collective electron oscillations, generating intense, tunable absorption/scattering for colorimetric sensing and enabling Surface-enhanced Raman scattering (SERS) via electromagnetic “hot spots” for trace analyte detection (19, 20). Critically, LSPR drives efficient light-to-energy conversion, underpinning AuNPs’ efficacy as potent photothermal agents and photosensitizers in photothermal therapy (PTT) and photodynamic therapy (PDT) (21). As the morphology progressively evolves, AuNPs’ amplified surface-area-to-volume ratio enhances biomolecular interactions, while exceptional electrical conductivity (>10^5^ S/m for 20-nm particles) facilitates ultrasensitive detection (18, 22, 23). LSPR “hot spots” also modulate fluorescence and enable fluorescence resonance energy transfer (FRET) (24).

AuNPs’ surfaces are readily functionalized via covalent conjugation, biomolecular assembly, or polymeric encapsulation (e.g., thiol anchoring, amide bonds, click chemistry, electrostatic adsorption) to confer targeting specificity, colloidal stability, and multifunctionality (25, 26). This enables active targeting (antibodies/peptides/aptamers) (27) or passive magnetic guidance (Fe_3_O_4_ composites) (28), allowing their use as multimodal contrast agents (MRI/X-ray/OCT) (29). Furthermore, AuNPs exhibit distinct catalytic activity in redox reactions, enabling applications in electrochemical biosensors (30). Collectively, these physicochemical properties underpin AuNPs’ transformative potential in diagnosing and treating gynecological malignancies.

Collectively, AuNPs represent a pivotal milestone in precision medicine, offering transformative potential for timely cancer intervention. The following sections detail the application of AuNPs, critically evaluating their contributions to diagnosing and treating gynecological malignancies to establish a reference framework for clinical practice.

## 2 AuNPs in cervical cancer

### 2.1 Diagnosis

#### 2.1.1 Colorimetric detection utilizing AuNPs

**Figure 1:**
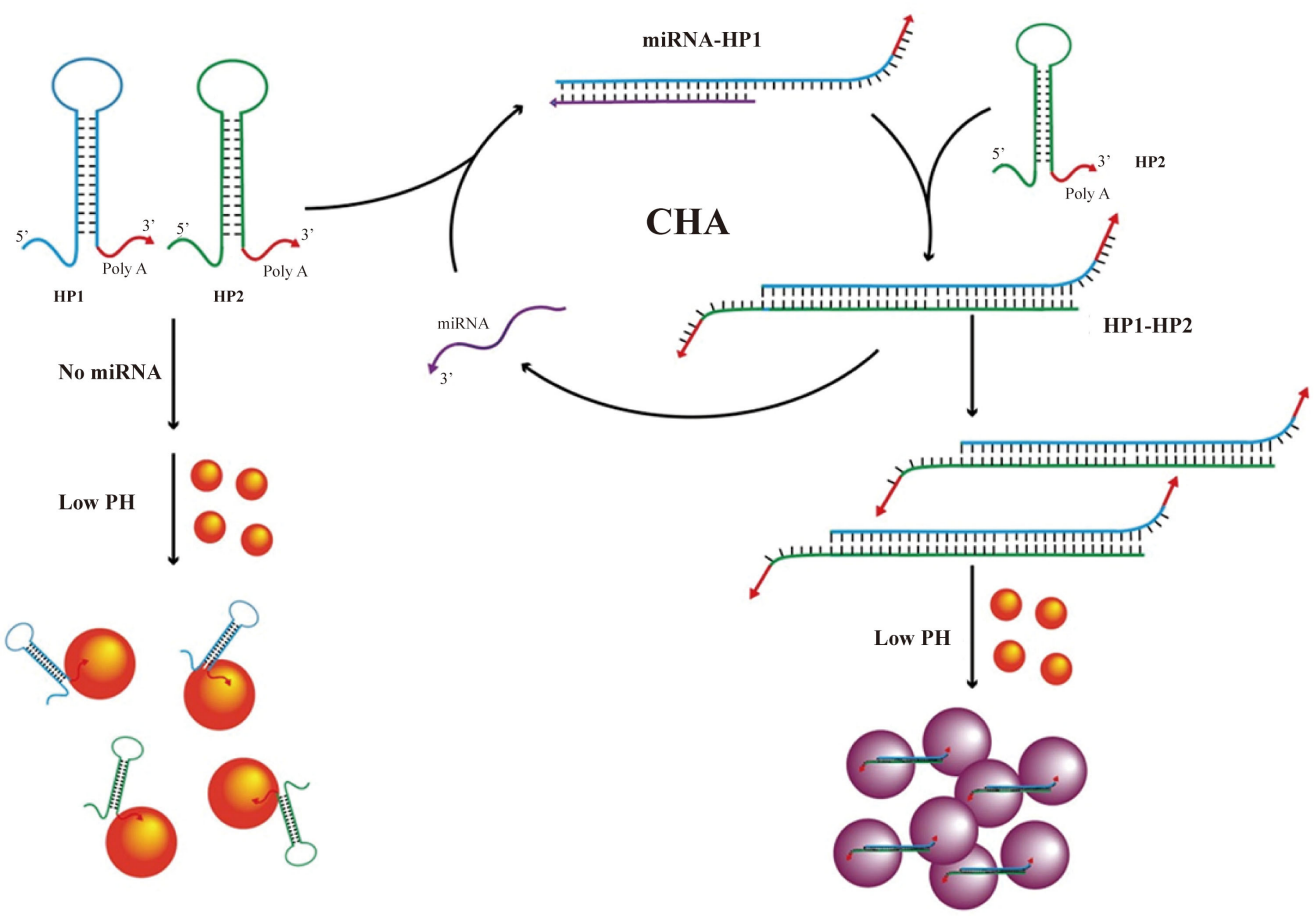
Schematic diagram of the detection process of smiRNA378 by colorimetric method based on AuNP design (31).(copyright permission obtained).

Functionalized AuNPs undergo LSPR peak shifts and visible color changes upon binding target molecules. AuNPs-based colorimetric assays thus exploit this phenomenon to assess levels of cervical cancer biomarkers through readily observable color transitions (31). microRNA-378 is consistently dysregulated in cervical cancer. Run and colleagues developed a colorimetric assay using catalytic hairpin assembly (CHA) and AuNPs for its detection (31). Target miRNA-378 triggers CHA between two hairpin probes, generating polymeric products. Multiple polyadenine blocks on these products adsorb and cross-link AuNPs (~13nm, Citrate-reduced) under acidic conditions, inducing aggregation. This shifts the LSPR, causing a visible color change from red to purple (**Figure 1**) (31). The assay leverages nucleic acid hybridization specificity, offering operational simplicity, high sensitivity, and strong specificity, with a LOD of 20.7 pM (31).

**Figure 2:**
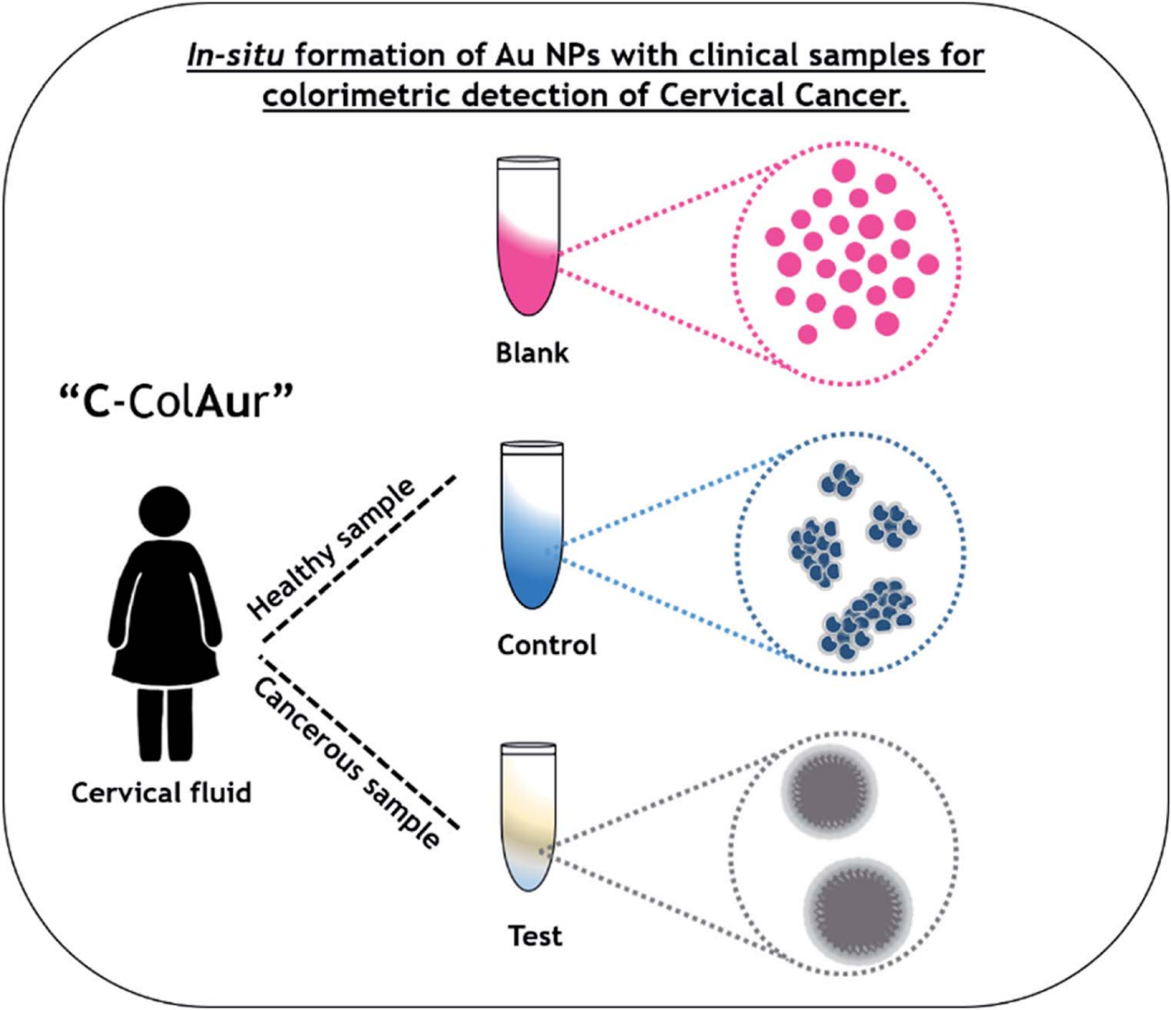
Schematic showing the colorimetric detection of cervical cancer using the label-free “C-ColAur” technique (33).

Motivated by the high cost of target-specific AuNP functionalization, label-free C-ColAur was developed as an alternative (32). Label-free C-ColAur is a nonspecific detection method leveraging AuNPs color shifts from LSPR. Target binding protects AuNPs from salt-induced aggregation, enabling rapid on-site pathogen screening via visible color changes, particularly in resource-limited settings (32). Applied to cervicovaginal fluid, it shows distinct color changes: AuNPs turn blue in healthy samples but remain unchanged in cancer samples, achieving high diagnostic accuracy (96% sensitivity, 87% specificity; **Figure 2**) (33). Transmission Electron Microscope (TEM) revealed significantly larger AuNP diameters (250~400nm, Citrate-reduced, Quasi-Spherical) in patients versus controls (15~30nm, Citrate-reduced, Quasi-Spherical), with reduced particle numbers and absence of aggregation in cancer samples (33). Tejaswini et al. proposed that cancer cell membrane components induce aggregation (34). Experimental validation confirmed that synthetic lipids, but not proteins or lipid-protein mixtures, replicated the color or spectral shift when reacted with HAuCl_4_·3H_2_O and ascorbic acid, indicating lipid-specific organization drives the mechanism (34).

HR-HPV is the primary cause of cervical cancer. Conventional detection (qPCR, isothermal amplification, dot blot) faces limitations including contamination risk, incomplete subtyping, and high cost (35). AuNP-based colorimetric assays (57.7 ± 4.2nm, Citrate-reduced, Quasi-spherical with dendritic protrusions) enable instrument-free, visual or smartphone-based readout of HPV-16/18, permitting quantitative results acquisition by untrained personnel without specialized equipmen (36). Carlos conjugated AuNPs (21.6 ± 0.1nm, Turkevich) with anti-HPV-16/18 L1 antibodies (37). Applied to 173 cervical samples, infection caused a color shift(red to purple) and LSPR red-shift (523~525 nm to 524~590 nm) (37). The assay detected HPV-16 L1 (linear range: 0.4~2.0μg/mL, LOD: 0.18μg/mL) and HPV-18 L1 (0.2~1.2μg/mL), identifying PCR-missed variants potentially via conserved L1 epitope recognition (37). To expand coverage, Jixue and team developed a multiplexed closed-tube PCR, detecting 17 HPV types (LOD: 0.5copies/μL, linear range: 0~1000copies/μL) with 99.05% accuracy (38).

#### 2.1.2 AuNPs-engineered electrochemical biosensors

Colorimetry lacks micro-scale sensitivity. Electrochemical biosensors convert biorecognition events to electrical signals (39). CEA, SCCA, Ki67, p53, and p16^INK4a^ are key cervical cancer biomarkers (35). Antibody-conjugated AuNPs enable their multiplexed detection. Electrochemical sensors universally use: 1) conductive substrates (Pt/Co/MoS_2_/WS_2_/rGO) to amplify AuNPs signals, and 2) engineered porous architectures (SiO_2_ dendrimers, multilayered nanoparticles, 3D networks) to enhance target capture and conductivity (**Table 1**) (40–42). These AuNPs-centered platforms show diagnostic promise (40). In a recent study, Hiranmoy et al. developed an ultrasensitive electrochemical immunosensor for cervical cancer biomarker p16^INK4a^ detection by modifying a glassy carbon electrode through sequential deposition of graphene oxide and ~70nm spherical AuNPs (43). The sensor utilized cysteamine-glutaraldehyde crosslinking to immobilize p16^INK4a^ monoclonal antibodies on the electrode surface (43). Compared to bare electrodes, Au/rGO-modified electrodes exhibited significantly enhanced peak currents with charge transfer resistance (Rct) reduced from 245 Ω to 14 Ω, demonstrating AuNPs’ capacity to facilitate electron transfer. Clinical serum analysis via square wave voltammetry achieved 100% accuracy with a detection limit of 167 fg/mL and linear range from 500 fg/mL to 100 ng/mL (43). The AuNP-engineered sensor demonstrated exceptional selectivity, storage stability, repeatability, reproducibility and regeneration capability.

**Table 1:** Research on the detection of serum tumor markers.

No	Particle size	Targe	Surface functionalization	Highlights of Study	Clinical sample	LOD	Linear range	Reference
1	317.4 ± 12.3nm	miRNA-21	Thiolation	MoS_2_/AuNPs composite-modified electrode and multilayered nanoneedle structure	Artificial serum	38aM	10aM~1uM	(41)
2	–	SCCA	Thiolation	Bimetallic PtCo nanoframe-modified electrode and dendritic DM-SiO_2_@AuPt core–shell nanostructures	Artificial serum	7.33fM	22.22fM~2.67µM	(42)
3	13.2 ± 2.32nm	p16^INK4a^; P53; Ki67	Thiolation; Electrostatic adsorption	Schematic of label-free sensing with redox reporters (Cd²^+^/DAP/MB);3SPCE array/GO/2D WS_2_/PEI-AuNPs/redox probe	Artificial serum	9.38nM; 5.49nM; 0.56nM	0.01~100ng/mL	(40)
4	~16nm	α2, 6-sialic acid	Electrostatic adsorption	ATR-FTIR combined with chemometrics for biosensor-cell interaction signal enhancement.	–	–	–	(44)
5	~75nm	p16^INK4a^	Thiolation	The Au-rGO material was employed for the first time to detect serum p16^INK4a^.	serum(15)	167fg/mL	500fg/mL~100ng/mL	(43)

**Figure 3:**
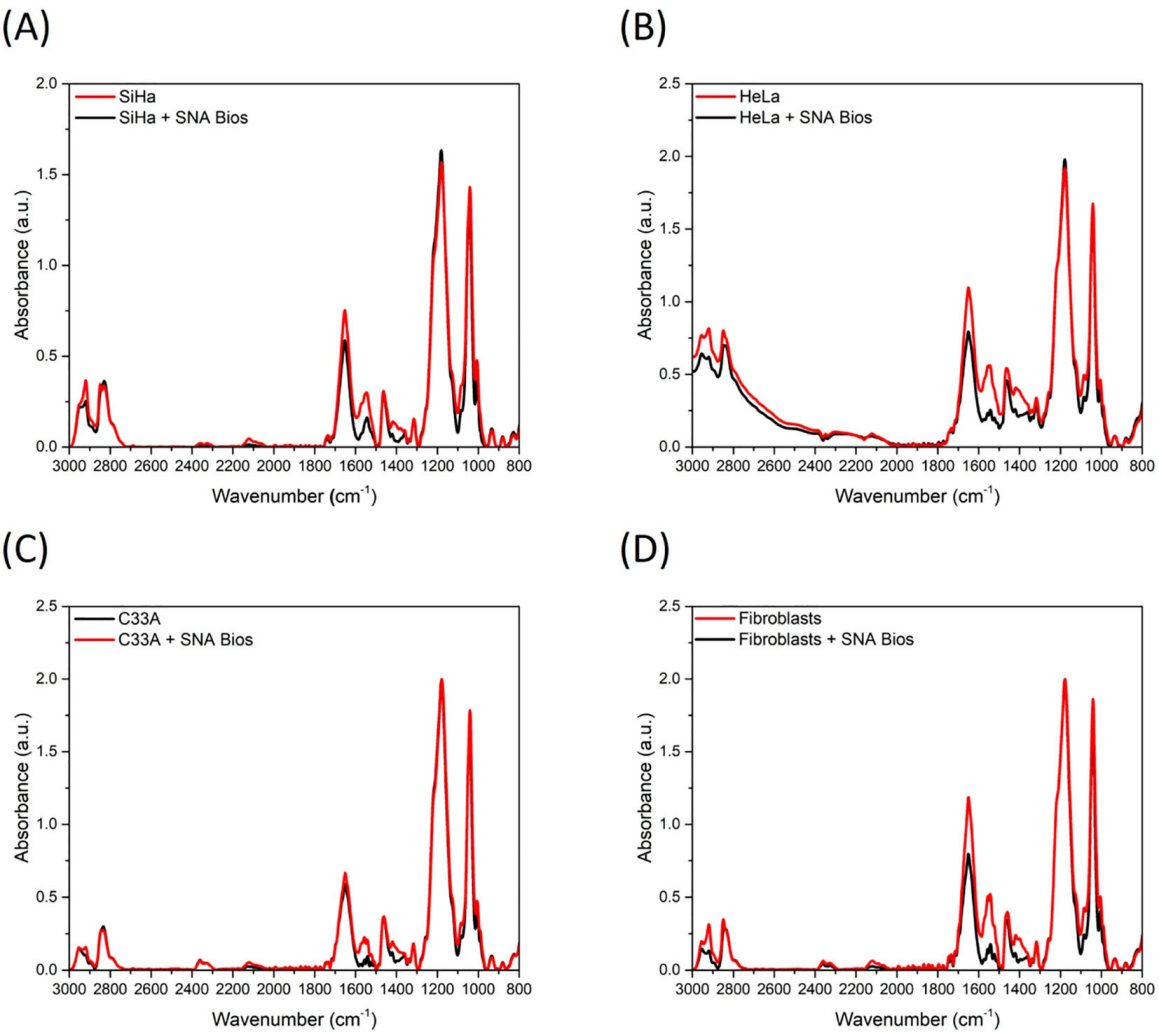
Mean ATR-FTIR spectra (baseline corrected and min/max normalized) of control cells and after interaction between SNA biosensors and **(A)** SiHa; **(B)** HeLa; **(C)** C33A; and **(D)** primary fibroblasts (44).

Similarly, while colorimetry detects cervical exfoliated cells, its accuracy suffers from subjective visual interpretation of subtle color shifts. AuNP-integrated (~16nm, Citrate-reduced, Spherical, chitosan-coated) photoelectric sensors enhance precision by targeting cancer-specific α2,6-sialic acid overexpression via SNA-conjugated AuNPs (44). Ricardo and team detected diagnostic spectral alterations at 1470, 1456, 1434, 1400, and 1350 cm^−^¹ in cancer-bound complexes using attenuated total reflection fourier-transform infrared spectroscopy (ATR-FTIR) (44). Notably, Ricardo et al. leveraged machine learning (ML), specifically principal component analysis (PCA), to identify sialic acid-associated spectral signatures in cervical cancer cell lines. Orthogonal principal components maximizing data variance were derived, enabling construction of confidence ellipse models that robustly discriminated primary fibroblasts from malignant cells (non-overlapping 95% confidence intervals; p<0.001) (44). This approach establishes a non-invasive diagnostic paradigm for early cervical cancer detection through surface-enhanced infrared absorption (SEIRA) biomarker profiling. (**Figure 3**).

AuNPs enhance HPV-16 DNA detection by increasing electrode surface area, accelerating electron transfer, and amplifying signals (39, 45). As **Table 2** shows, varied electrode designs achieve LODs<0.2fM (46–49). Dielectric and photoelectrochemical sensors offer enhanced precision, with maintained functionality during long-term cold storage (46, 48).

**Table 2:** Recent advances in HPV detection biosensors.

No	Particle size	Targe	Surface functionalization of AuNPs	Highlights of Study	Clinical sample	LOD	Linear range	Reference
1	–	HPV 16 DNA	–	Exhibits strong anti-interference capability in human serum	Artificial serum	0.5475 fM	100fM~1000nM	(50)
2	–	HPV-18 DNA	Thiolation	rGO-MWCNT-AuNPs nanocomposite-functionalized SPCEs	Cervical smear	0.05fM	0.01fM~0.01nM	(47)
3	16 ± 1nm	HPV-16 DNA	Thiolation	APTES-modified GCE; CP-AuNPs immobilized via phosphate-amine electrostatic adsorption	Artificial serum	0.1731fM	100fM~10uM	(46)
4	18.9 ± 1.5nm	HPV-16 E6/E7 mRNA	Thiolation; Covalent bonding	LAMP-amplified mRNA; Streptavidin modification; ssDNA-AuNPs targeting; SA-HRP signal amplification	Cervical smear(20)	0.08fM	100nM~100uM	(48)
5	–	HPV L1	Electrostatic adsorption	PANI electrode with AuNPs deposition; MY11 degenerate probe	Cervical smear	0.113nM	1~100 pg/μL	(51)
6	~5nm	HPVs	–	Screen-printed photoelectrode array modified with TiO_2_@AuNPs	Positive cervical smear(20); negative cervical smear(20)	0.1copies/μL	0.6~600copies/μL	(52)
7	9 ± 1.5nm	HPV-18 DNA	Covalent bonding	SiO_2_-micro-IDE substrate; APTES-AuNPs dehydration-condensation modification	Cervical smear(20)	0.529aM	1aM~100fM	(49)
8	~40nm	HPV-16/18 DNA	Electrostatic adsorption	CuCo_2_S_4_-ZnIn_2_S_4_-S heterojunction interface; Paper-film composite chip	Serum	0.21pM; 42.92pM	–	(53)
9	~15nm	HPV-16	–	Using SynSed technology as an alternative method for particle transfer in DFM imaging	Artificial serum	10fM	0pM~500pM	(54)
10	~50nm	HPV-16	Covalent bonding	This is the first dual-colorimetric strategy based on AuNPs for detecting double-stranded HPV-16 viral genome.	Cervical smear	1.9nM	0.45~6.72nM	(45)

**Figure 4:**
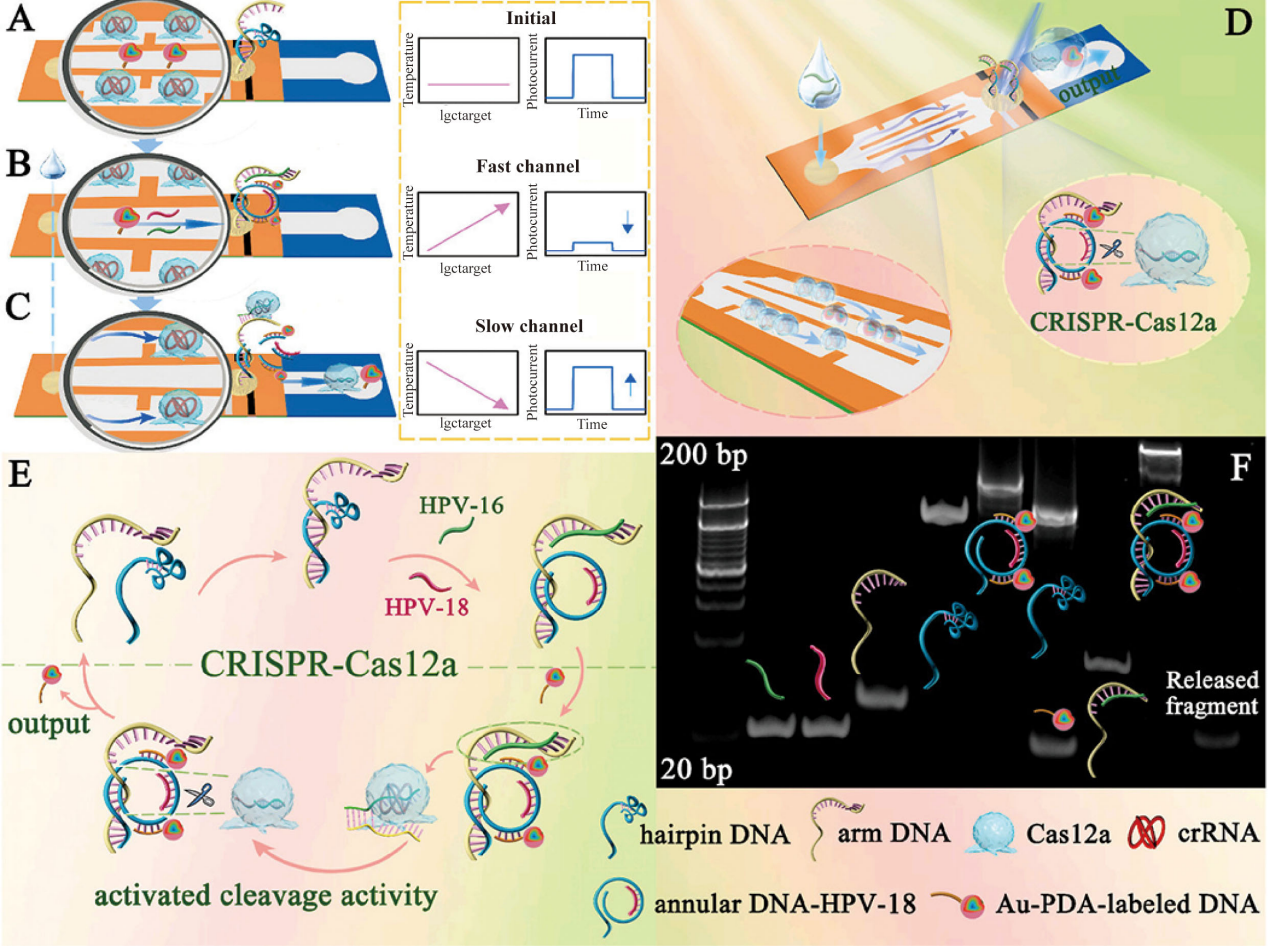
Detection steps and interactive signal change process in the **(A)** initial state, **(B)** the DNA conformational change state, and **(C)** CRISPR-Cas12a activation state. **(D)** Schematic diagram of the proposed lateral flow biosensor. **(E)** Signal amplification process of the CRISPR-Cas12a-driven dual-readout lateral flow biosensor. **(F)** Feasibility analysis for the self-assembly process of the annular DNA and trans-cleavage instinct of CRISPR-Cas12a (53). (copyright permission obtained).

Lin and colleagues’ paper-based sensor achieves significant cost reduction (<$1.00), miniaturization, and commercial viability (53). Utilizing lateral flow principles, HPV-16/18 DNA hybridizes with probes in a rapid-flow zone, forming circular structures that bind AuNPs (~40nm, Spherical)-polydopamine (PDA). Light irradiation triggers AuNP-mediated photothermal conversion, intensifying thermal response while diminishing photocurrent. Subsequent fluid migration to a slow-flow zone enables CRISPR-Cas12a-mediated DNA cleavage and HPV fragment release, reversing signals (**Figure 4**) (53). This system detects HPV-16/18 DNA within 30 minutes with high specificity (LOD: 0.21pM and 42.92pM, respectively) (53). However, Lin’s sensor is susceptible to temperature, salt concentration, and reaction time variations due to pre-hybridization nucleic acid cleavage. These factors exacerbate electrical signal transduction limitations, including single-molecule counting inability. To overcome this, Jia and team integrated dark-field microscopy (DFM) imaging, circumventing signal dependency while employing synergistic sedimentation (SynSed) to minimize nanoparticle diffusion (~15nm, Citrate-reduced) (54). This optimized system achieves a LOD of 10 fM.

Beyond HPV-16 and HPV-18, other high-risk HPV types (31, 56, 59, 68) also contribute to cervical cancer development (35). To address this, platforms incorporating MY11 probes targeting conserved regions of the HPV L1 gene have been integrated with AuNPs-doped detection systems. AuNPs amplify current-voltammetric signals generated by minor nucleotide variations within the L1 conserved regions of HPV-16/31/33/45/58, enabling the construction of HPV genotyping profiles (51). Conversely, an alternative strategy anchors multiple recognition probes within a photoelectrochemical biochip array (PEBA) platform composed of TiO_2_@AuNPs composites (52). This highly integrated chip-based format achieves an exceptionally low LOD of 0.1 copies/μL, demonstrating high concordance with clinical results (52).

#### 2.1.3 SERS detection based on AuNPs

**Figure 5:**
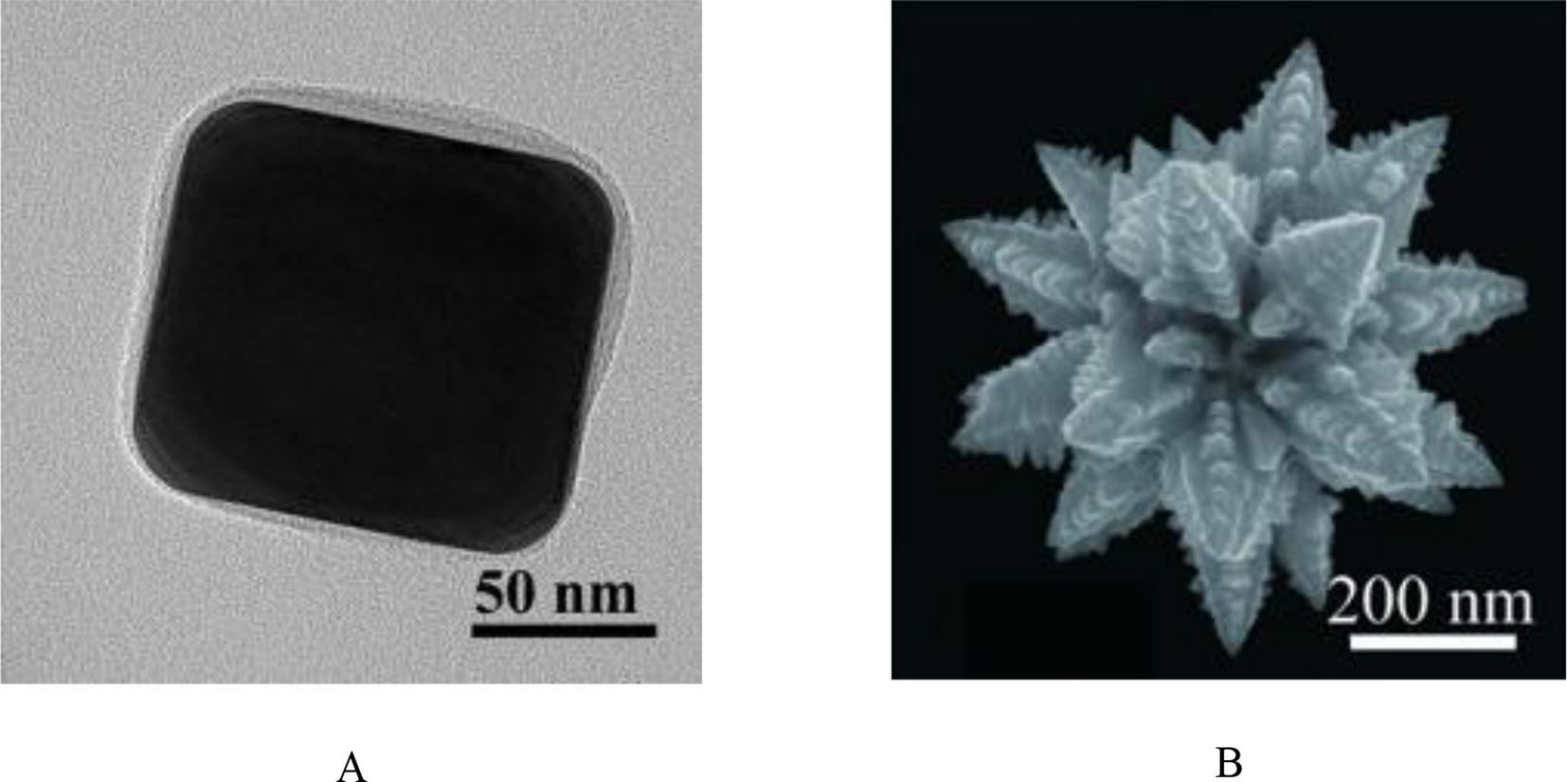
**(A)** High magnification SEM image of Cube-AuNPs (58); **(B)** High magnification SEM image of AuNF (55). (copyright permission obtained).

SERS surpasses electrochemical biosensors in AuNPs-based serological diagnostics by circumventing electrode constraints. As **Table 3** shows, platforms utilize: 1) sharpened AuNPs geometries to intensify electromagnetic hotspots, and 2) ordered array chips to enhance spectral reproducibility (55–58). However, significant LOD variability exists (e.g., 191.73 fM vs. 10 fM for SCCA in serum) (55, 58), likely stemming from AuNPs morphology and Raman reporter differences (**Figure 5**).

**Table 3:** Research on the detection of cervical cancer (SERS).

No	Particle size	Targe	Surface functionalization of AuNPs	Highlights of Study	Clinical sample	LOD	Linear range	Reference
1	~600nm	SCCA; OPN	–	Ag-AuNFs bimetallic nanocomposites with arrow-headed tip nanostructures	Serum(150)	191.73fM; 132.97fM	10 pg/mL~9 μg/mL	(55)
2	~45nm	B7-H6	Covalent bonding	Zwitterion-modified stealth sensor with pH-stable spiky AuNPs	Positive serum(9); negative serum(1)	10fM	10fM~10nM	(56)
3	~20nm	CpG methyltransferase	–	Site-selective assembly of AuNP arrays on monolayer SiO_2_ arrays yielding Au@SiO_2_ array substrates;RCA signal amplification strategy	Serum(150)	0.251mU/mL	0.005~50U/mL	(57)
4	~20nm	SCCA; CEA	–	Cubic Ag-Au bimetallic nanoparticles integrated on Au@SiO_2_ array substrates;Pump-free flow via capillary pump/hydrophilic-treated channels;Multichannel parallel auto-detection	Positive serum(60); negative serum(30)	10fM; 2fM	1pg/mL~1μg/mL	(58)
5	70~80nm	p16;Ki-67	Covalent bonding	AuNS@Ag nanoflowers enable 20-min slide detection, bypassing ICC staining.	Cervical smear	–	–	(59)
6	~20nm	miRNA21/124/143	–	SPRi: AuNP-helper probes enhance chip signals; SERS: Triplex Raman reporters hybridize with target miRNAs.	–	1 fM; 0.8 fM; 1.2 fM	10 fM~100pM	(60)
7	40~45nm	–	–	SERS on single cells/spheroids/DNA + chemometrics (PCA/LDA/SVM) for cervical cancer detection; validated by cytology/HPV PCR/UFLC metabolomics.	Cervical smear	–	–	(61)
8	~80nm	–	–	“Hotspot”-rich 3D plasmonic AuNP-nanomembranes: LLISA-assembled monolayers triple-stacked on ITO; optimized with AI.	Positive serum(12); negative serum(5)	–	–	(62)
9	~25nm	–	–	A reliable detection protocol was established to obtain highly stable and reproducible serum SERS spectra	Positive serum(12); negative serum(24)	–	–	(63)
10	83 ± 6nm	sEV	–	Detection of cancerous exosomes using plasmonic AuNPs nanosheets as SERS substrates combined with CD63 nanoflares	Positive serum(11); negative serum(8)	4.7 × 10^5^ particles/mL.	1 × 10^6^~2 × 10^8^particles/mL	(64)

SERS and Surface Plasmon Resonance Imaging (SPRi) are increasingly favored for cervical cancer biomarker detection (**Table 3**). Yifan and team developed a dual-mode SPRi/SERS biosensor with a polyA capture probe/target miRNA/AuNPs-enhanced probe sandwich architecture (60). This platform concurrently detects miRNA-21/124/143 on a single chip (60). Target hybridization forms ternary complexes where AuNPs enhance SPRi via refractive index modulation while generating SERS hotspots through LSPR (60). Dual-signal redundancy reduces false positives (serum recovery: 90.0–100.2%) (60). This high-throughput platform provides dual-verified nucleic acid detection with clinical utility.

The spatial arrangement of AuNPs in SERS sensors critically determines analytical outcomes. Xingkang et al. engineered a nanoscale monolayer film of uniform AuNPs (83 ± 6nm) to generate evenly distributed dense hotspots, overcoming the random hotspot distribution characteristic of conventional plasmonic colloidal solutions (64). This design prevented heterogeneous electromagnetic signals arising from nanoparticle aggregation while enhancing SERS signal reproducibility and sensitivity (64). Furthermore, integration of CD63 nanoflares with AuNPs created supplemental hotspots that amplified SERS intensity by 4.1-fold (64). The resulting platform exhibited linear detection of cancer exosomes from 1×10^6^ to 2×10^8^ particles/mL with a limit of detection (LOD) of 4.7×10^5^ particles/mL (64).

Artificial intelligence enables more efficient mining of the massive data generated by AuNP probes in SERS sensing analysis, forming the foundation for low-cost nonspecific analysis. Hongmei and team established that 10 ppm serum prevents AuNPs encapsulation while enhancing dispersion homogeneity (63). After screening lasers, they selected 785 nm and 633 nm excitation (excluding 532 nm due to fluorescence interference), achieving 0.9609 spectral cosine similarity at 10 ppm (63). Clinical validation with 36 sera (24 healthy/12 cancer) identified differential peaks at 1201 cm^−^¹ and 1312 cm^−^¹. PCA achieved complete group separation, establishing the first standardized SERS reference library for cervical cancer sera. This methodology provides a robust foundation for clinical SERS translation (63).

Meanwhile, this simultaneously enables unsupervised recognition of cervical cell pathology through AI analysis of non-functionalized gold nanoparticles (nfGNPs). Karunakaran et al. implemented support vector machine (SVM) algorithms to achieve high-precision classification of NRML/HSIL/CSCC specimens (94% ± 0.73% accuracy for single-cell analysis), markedly improving post-PCA specificity with ROC-validated diagnostic progression (AUC >0.98) for cervical lesion stratification (61). In contrast, Diao et al. adopted a PCA-LDA fusion strategy (62). Following Savitzky-Golay filtering with airPLS background correction and min-max normalization, PCA reduced spectral dimensions to 46 principal components retaining 95% of the variance (62). LDA then compressed these into two discriminative factors. This hierarchical approach resolved non-overlapping 95% confidence ellipses observed under singular methods and attained 91.1% accuracy in differentiating exosomes across H8, HeLa, and MCF-7 cell lines (62). The model demonstrated robust diagnostic efficacy, with AUCs ranging from 0.93 to 0.99, and maintained 93.3% overall accuracy in clinical validation (62). It perfectly classified breast and cervical cancer serum exosomes while correctly identifying 80% of healthy samples. This established a non-invasive framework for SERS-driven early cancer detection (62).

#### 2.1.4 AuNPs-enabled innovative imaging strategies

Imaging is vital for non-invasive cervical cancer diagnosis. Tumor-targeting ligand-functionalized AuNPs (e.g., folic acid, oligotyrosine/RGD/NLS peptides) serve as effective contrast agents (65). The tumor microenvironment further enhances AuNPs (50.3 ± 1.1nm, Citrate-reduced, spherical) accumulation, where acidic pH triggers hydrolysis of citraconic anhydride linkages in AuNPs-doxorubicin (DOX) complexes (66).

**Figure 6:**
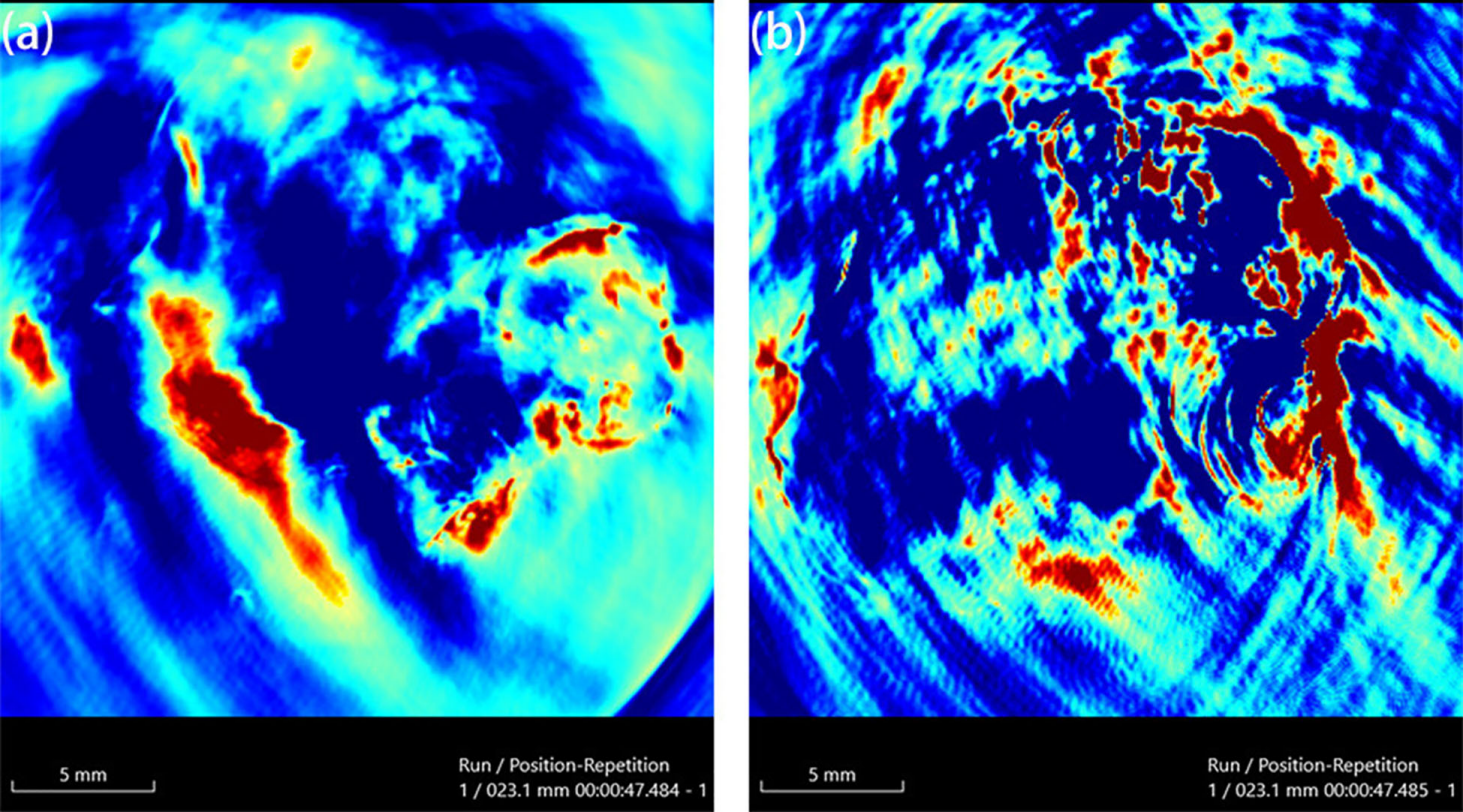
PAI of before **(a)** and after **(b)** injection 200 µg/mL of Au-UCNPs-DSPE-PEG2K in Balb/c mice (67).

Radioiodinated AuNPs serve as contrast agents for clinical imaging (MRI/US/CT/PET) (29). Min and colleagues engineered pH-responsive theranostic AuNPs (50.3 ± 1.1nm, Microwave-assisted synthesis, spherical) with PEGylated surfaces and citraconic anhydride-linked DOX (66). At pH 5.5, linker hydrolysis triggers DOX release and electrostatic aggregation (35-nm UV-Vis redshift; TEM-confirmed), amplifying imaging signals (66). Radioiodination achieved 35.4% labeling efficiency, with PET showing 1.37%ID/g tumor uptake at 2h and >38% cancer cell binding (66). Photoacoustic imaging (PAI) generates biological images by detecting photon emissions from contrast agents. AuNPs exhibit exceptional PAI properties due to high photothermal conversion efficiency (66). The Au-UCNP-PEG_2k_ nanocomposite serves as both photodynamic therapy agent and multimodal imaging platform (~5nm, Hydrothermal method, spherical) (67). *In vivo* studies show superior PAI performance under near-infrared region (NIR) irradiation compared to bare AuNPs, functioning as a trimodal contrast agent for comprehensive tumor diagnostics (**Figure 6**) (67).

AuNPs enhance lesion identification in laboratory imaging (68). Under DFM, FA-targeted AuNPs@Ag@AgI nanostructures internalized by HeLa cells enable precise spatial localization via amplified light scattering (68). Simultaneously, AuNPs’ plasmonic properties mediate surface energy transfer to fluorescent donors (e.g., TAP, PHEN), inducing controlled fluorescence quenching within cells, supporting development of real-time imaging agents (68).

### 2.2 Treatment

#### 2 .2.1. AuNPs-based drug delivery systems

AuNPs enable targeted cancer cell eradication via drug and ligand conjugation. Therapeutic payloads encompass Methotrexate (MTX) (69), DOX (66), Paclitaxel (PTX) (70), and luteolin (71). Common cervical cancer targeting ligands comprise Folic Acid (FA), Hyaluronic Acid (HA), and AS1411 aptamer (**Table 4**). Emerging agents show complementary potential (72). The investigational compound IQ activates TLR7/8-mediated immune responses and NF-κB-dependent apoptosis. When complexed with nucleolin-targeting AS1411 aptamer on AuNPs (IQ-AS1411-AuNPs), MTT assays demonstrated significantly reduced HeLa cell viability (72).

**Table 4:** Design of AuNPs for the treatment of cervical cancer (drug delivery).

No	Particle size	Surface functionalization of AuNPs	Drug	Highlights of Study	Cell line	Toxic	Reference
1	~11nm	Thiolation	MTX	AuNPs with FA modification; High MTX loading (>98.7% EE); GSH-responsive MTX release	U-87MG; HeLa; A549; PC3; HEK-293	The MTT results indicate no toxicity.	(69)
2	50.3 ± 1.1nm	Thiolation; Covalent bonding	DOX	Citraconic anhydride linker enables pH-responsive DOX release in tumor microenvironment	HeLa	Considered	(66)
3	~20nm	Thiolation	PTX	One-pot synthesis using PTX as dual reductant/stabilizer; Eco-friendly process without additional chemicals	SiHa; HT-29	Considered	(70)
4	18.30~21.16nm	Thiolation	Imiquimod	AS1411-mediated selective drug delivery; Enhanced vaginal tissue retention for gynecological cancer therapy	HeLa; HEC-1-A; NHDF; Franz	No toxicity	(72)
5	26.35 ± 2.13nm	Thiolation; Covalent bonding	DOX	pH-responsive DOX release; Dose-dependent cytotoxicity in HeLa cells; Synergistic therapeutic platform for ovarian cancer	HeLa	A dose-dependent toxicity was observed.	(73)

Beyond ligand-mediated targeting, tumor microenvironment (TME) characterized by hypoxia, low pH, elevated GSH, and ROS, directs AuNPs accumulation. A TME-responsive platform bypasses ligand functionalization, exemplified by GSH-cleavable disulfide-linked AuNPs (~11nm, Brust-Schiffrin, spherical) achieving 98.7% MTX encapsulation for cervical cancer (69). Similarly, DOX-conjugated AuNPs utilize pH-labile hydrazone bonds that hydrolyze selectively in acidic TME conditions, enabling clinically translatable targeted therapy (66, 73).

**Figure 7:**
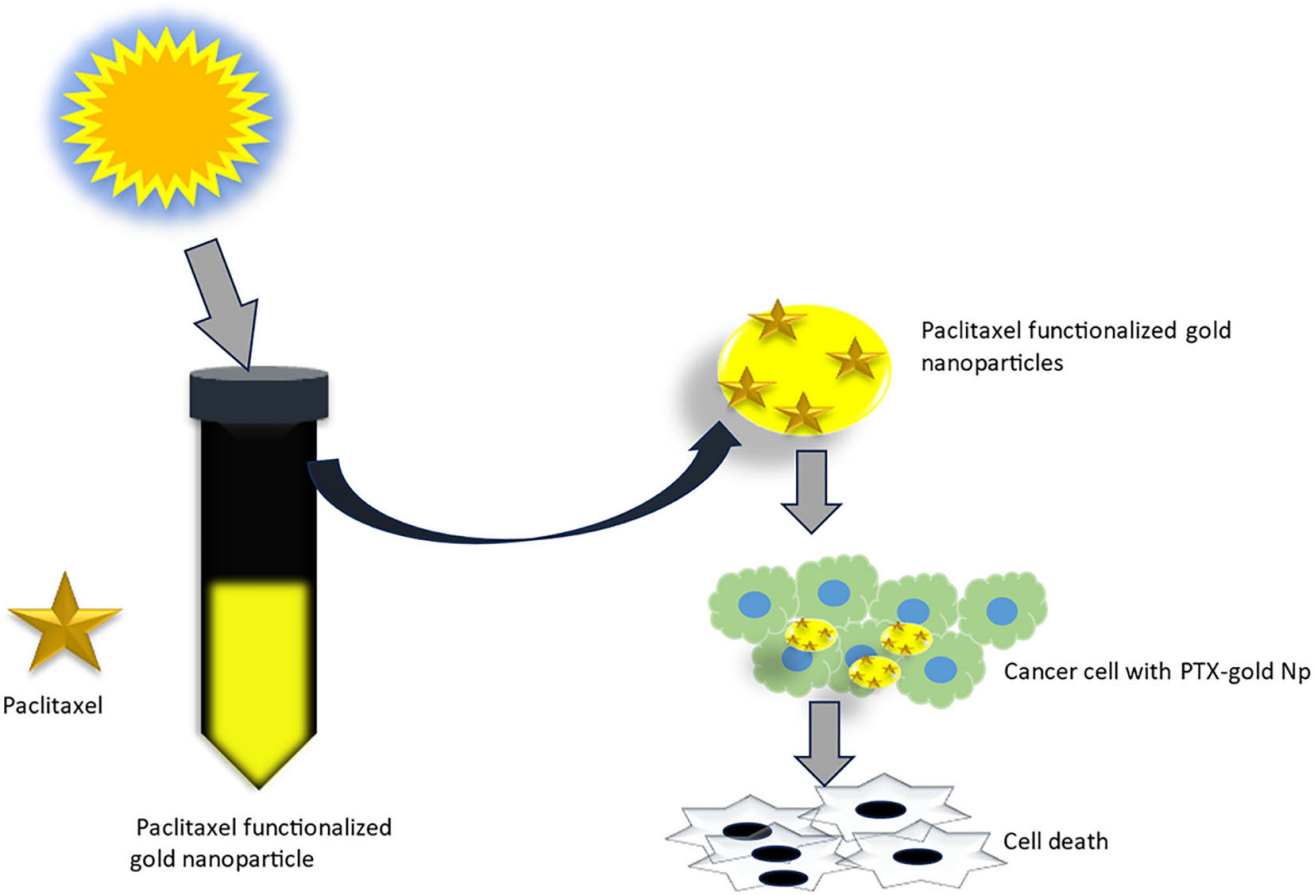
One pot synthesis of paclitaxel functionalized gold nanoparticles and their anticancer studies (70). (Copyright permission obtained).

Toxicity and complexity concerns around chemical stabilizers (e.g., 3MPA/3MPS/PVP) drove Kamini et al. to develop a novel PTX delivery platform (70). Their method synthesizes PTX-AuNPs in one step by adding silver ions to PTX/gold ion suspensions under sunlight (**Figure 7**), where PTX acts as both therapeutic payload and reducing/stabilizing agent (70). This eliminates pre-synthesized AuNPs and exogenous stabilizers, reducing costs while bypassing conventional drug-loading, though the photochemical mechanism requires further study (70).

#### 2.2.2 Intrinsic antitumor effects of AuNPs

AuNPs exert intrinsic cytotoxicity against cervical cancer cells via apoptosis modulation (72, 74). Concentration-dependent upregulation of pro-apoptotic markers (BAX, p53) and downregulation of anti-apoptotic factors (Bid, BCL-2) occur with concomitant caspase activation (75). This cascade initiates from AuNPs-induced intracellular ROS elevation, confirmed by flow cytometry showing increased cytoplasmic ROS and mitochondrial superoxide production (76). AuNPs generate ROS partly through intrinsic catalytic activity, converting H_2_O_2_ into H_2_O and O_2_ while enhancing catalase activity to modify the tumor microenvironment via hypoxia alleviation and immune potentiation (77). Additionally, AuNPs elevate superoxide dismutase (SOD) activity, causing H_2_O_2_ accumulation that activates caspase-dependent signaling pathways to induce cancer cell apoptosis (77). AuNPs’ cytotoxicity against cervical cancer cells depends critically on nanoparticle diameter and subcellular localization (78). Dae et al. found extracellular monodisperse AuNPs prolong HeLa cell mitosis without inducing apoptosis by forming division-disrupting nano-barriers. By contrast, 111nm extracellular AuNPs trigger ROS-mediated cell death with cytokinesis arrest, while 83nm particles cause transient M-phase delay followed by normal cytokinesis and G_1_-phase entry (78).

#### 2.2.3 High-efficiency photothermal tumor ablation utilizing AuNPs

AuNPs act as efficient photothermal transducers, enabling precise tumor ablation under targeted guidance (21). Recent refinements include S-nitrosothiol-modified AuNPs releasing nitric oxide during PTT to simultaneously soften tumor extracellular matrix and generate mitochondrial reactive nitrogen species for enhanced cytotoxicity (79). Multibranched gold nanocrystals further outperform spherical or rod-shaped counterparts in photothermal conversion efficiency, confirming morphology as critical design parameter (21). PDT employs photosensitizers to generate cytotoxic ROS but faces limitations including toxicity and poor cellular uptake. Armin and team further demonstrated that AuNPs functionalized with protoporphyrin IX and FA via mercaptohexanol linkers enhance both cancer selectivity and cytotoxic efficiency (80).

#### 2.2.4 Radiosensitization effects of AuNPs in radiotherapy

AuNPs enhance cervical cancer radiotherapy efficacy through their high atomic number (Z = 79), which dramatically exceeds biological elements. This promotes X-ray/γ-ray absorption, generating amplified secondary electron fluxes that directly damage tumor DNA (81). Radiation-activated gold nanorods (AuNPs, 54.6 ± 7.11nm, spherical) further catalyze water decomposition, producing cytotoxic ROS (82). Functionalized variants concentrate radiation energy within lesions. Radiosensitization varies significantly with geometry: AuNRs surpass spheres (83). Nanocubes deposit 18.5% (18 MV) to 23.1% (6 MV) higher electron doses than nanospheres within 1.1 µm radii, achieving maximum dose enhancement at 6 MV (84).

## 3 AuNPs in ovarian cancer

### 3.1 Diagnosis

#### 3.1.1 Colorimetric detection utilizing AuNPs

Colorimetry enables rapid ovarian cancer biomarker screening but suffers from limited resolution. Eda et al. circumvented this via smartphone-integrated analysis leveraging mobile imaging hardware advances (~40nm) (85). Alternatively, Hao et al. engineered Mg/Fe-layered double hydroxide nanoflowers as high-density AuNPs (~10nm, Rapid injection synthesis) carriers for lateral flow immunoassay (LFIA) (86). These porous templates achieve ultrahigh AuNPs loading, lowering HE4 detection to 50 pM (86).

#### 3.1.2 AuNPs-engineered electrochemical biosensors

CA125 is the clinical gold standard for ovarian cancer serology. Current AuNP-based detection platforms vary primarily in electrode composition and surface modification strategies(**Table 5**), integrating AuNPs with carbon nanomaterials (graphene, CNTs), polymers (chitosan, polydopamine, PAMAM), or novel frameworks (MXenes, MOFs) to enhance nanoparticle density, antigen capture efficiency, and electron transfer kinetics. The integration of MOFs with AuNPs achieves reproducible CA12-specific recognition, demonstrating a RSD of 2.98% in repeatability experiments (87). Zahra’s MXene/GQD/AuNPs composite achieves a record LOD of 0.075nU/mL (88). DLS sensors offer the broadest linear range (5fg/mL~50ng/mL) for high-dynamic CA125 quantification (89), while AuNP-DNA fluorescence quenching enables continuous biomarker monitoring. Near-field communication (NFC) integration further streamlines data acquisition, enhancing clinical utility (90).

**Table 5:** Sensor design scheme for detecting CA125 based on AuNPs.

No	Particle size	Surface functionalization of AuNPs	Highlights of Study	Clinical sample	LOD	Linear range	Reference
1	–	–	Cu single-atom/AuNPs modified electrode	Artificial serum	0.37 pg/mL;1.58 pg/mL	0.005~500 ng/mL	(91)
2	~14nm	Thiolation; Covalent bonding	PAMAM/AuNPs and 3D rGO-MWCNTs modified electrode;Succinic anhydride-modified chitosan	Artificial serum	6 μU/mL	0.0005~75 U/mL	(92)
3	~15nm	Thiolation	*In-situ* grown AuNPs/GaN Schottky junction via H_2_O_2_ etching;AuNPs size-controlled Fermi level/charge transfer efficiency	Serum(4)	0.3 U/ml	1~100 U/mL	(93)
4	~13nm	–	Target-aptamer binding modulated AuNPs aggregation for fluorescence on/off;	Serum(4)	0.015 U/mL;7.5 pg/mL	0.01~2.0 U/mL;0.01~0.9 ng/mL	(94)
5	~13nm	Thiolation	Dual-signal detection: DLS particle size and fluorescence;	Serum(2)	1.1 fg/mL	5 fg/mL~50 ng/mL	(89)
6	–	Thiolation; Covalent bonding	Microporous carbon modified SPCE;Smartphone-based NFC signal acquisition;	Serum(6)	0.4 U/mL	0.5~50.0 U/mL	(90)
7	~4.5nm	–	TDN-enhanced TMSD with AuNPs/Ru/ZIF-MOF signal probes;	Serum(4)	0.006 pg/mL	0.01 pg/mL~10 ng/mL	(95)
8	122 ± 11nm	Thiolation	AuNPs modified FTO electrode;Oligonucleotide recognition elements (antibody-free);	Serum(3)	2.6 U/mL	10~800 U/mL	(96)
9	–	–	MXene-GQD/AuNPs modified electrode	Artificial serum	0.075 nU/mL	0.1 μU/mL~1 U/mL	(88)
10	–	Thiolation; Covalent bonding	AuNPs/RGO/PTH-modified DSPCE electrode	Artificial serum	0.069 pg/mL; 0.058 pg/mL	1~100 pg/mL	(97)
11	~70nm	–	AuNPs and DES-synthesized PTB co-modified SPCE	Artificial serum	1.20 pg/mL	5~100 pg/mL	(98)
12	–	–	Electrodes modified with MOF@AuNPs-based nanocomposites	Serum	7.185nU/mL	10~70nU/mL	(87)
13	–	Thiolation; Electrostatic adsorption	Two biocompatible 2D COFs (EP-TD-COF and AuNPs@COFBTT-DGMH) effectively preserved antibody activity and provided a favorable microenvironment, synergistically enhancing the stability and sensitivity of the immunosensor	Artificial serum	0.089mU/mL	0.00027~100U/mL	(99)
14	~54.61nm	–	The hybrid nanostructure of α-MnO_2_ nanorods and AuNPs enhanced conductivity and sensitivity	Serum	9.82ng/mL	10~70ng/mL	(100)
15	–	–	CuCo-ONSs@AuNPs nanocomposite-modified electrode	Serum(6)	39nU/mL	0.1μU/mL~1mU/mL	(101)
16	–	Thiolation	A biosensor capable of simultaneously interacting with sEV and CDDP was developed, enabling the simultaneous quantification of sEV and CDDP using SERS, thereby overcoming the heterogeneity and protein interference issues in SERS analysis of sEV	Positive serum(99); negative serum(20)	–	–	(102)

**Figure 8:**
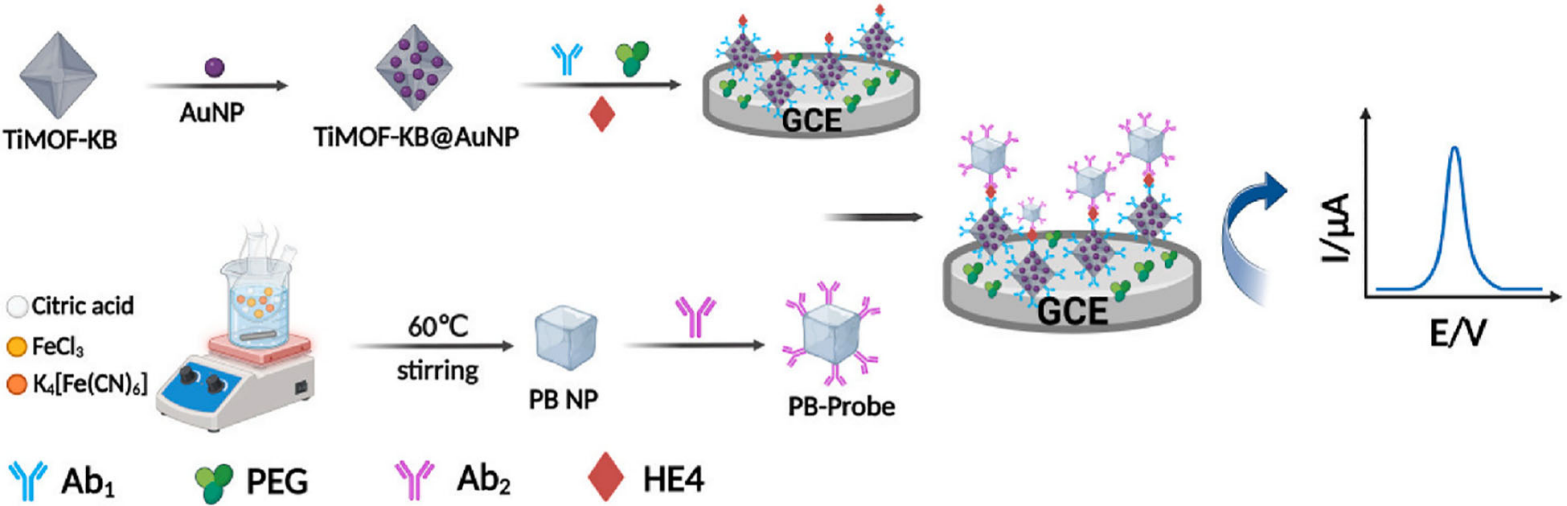
Shows the principle of the prepared electrochemical biosensor for fast detection of HE4 (107). (Copyright permission obtained).

Beyond CA125, AuNP-based sensors target biomarkers including p53, HE4, exosomes, and DNA methylation (91, 103–106). Following Weiwei et al.’ s MOF-AuNPs for CA125 (95), Xu and team developed a sandwich electrochemical immunosensor using synergistic signal amplification between Prussian blue (PB) and thiolated ionic MOF composites (TIMO^+^F-KB@AuNPs) for HE4 quantification (107). The platform employs TIMO^+^F-KB@AuNPs as conductive sensing substrates for capture antibody (Ab_1_) immobilization, while PB nanoparticles carry detection antibodies (Ab_2_). HE4 binding forms an Ab_1_-HE4-Ab_2_-PB complex, generating electrochemical signals proportional to concentration (linear range: 0.1~80ng/mL, LOD: 0.02ng/mL) (107). Demonstrating high selectivity, reproducibility, and stability with 97.10~114.07% serum recovery, it enables early ovarian cancer diagnosis (**Figure 8**). Separately, superparamagnetic CoFeB enhances AuNPs-based p53 detection (LOD: 0.006 U/mL) (104). Furthermore, exosomes predict ovarian cancer chemotherapy response. Meshach and team developed a cysteine-functionalized AuNP (Au-cys) biosensor using SERS to simultaneously capture cisplatin and small extracellular vesicles from biological samples, enabling concurrent early detection and treatment efficacy assessment (102).

AuNPs facilitate ovarian cancer diagnosis via urine, exhaled gas, and cyst fluid analysis (108, 109). Thomas and team pioneered nanopore sensing where AuNPs capture 13 cysteine-containing urinary peptides within α-hemolysin nanopores, generating distinct current signatures: stepwise reductions for small peptides versus polymorphic fluctuations for larger ones, enabling prolonged single-molecule characterization (108). AuNPs-based platforms also detect microbiome-derived Volatile organic compounds (VOCs) in exhaled breath with 82% screening accuracy (110).

#### 3.1.3 AuNPs-enabled innovative imaging strategies

AuNPs drive transformative ovarian cancer imaging advances (19). Dheeraj and team engineered FA-targeted, hydrazinonicotinamide-chelated AuNPs enabling efficient 99mTc radiolabeling for tumor-specific single-photon emission computed tomography (SPECT) contrast (111). Further functionalization yielded GO/SPIO/AuNP composite sheets integrating PTT, radiotherapy, and MRI within a unified theranostic platform (112).

MARS spectral photon-counting CT (SPCCT), though primarily preclinical, enables material-specific imaging via energy-dependent X-ray attenuation. Dhiraj et al. functionalized AuNPs with LHRH via PEG tethers for ovarian cancer targeting (113). Intraperitoneal administration in murine models achieved selective accumulation in peritoneal tumors (0.46~2.12ng/mg, ICP-MS). While current SPCCT sensitivity limited absolute quantification, it mapped gold distribution patterns (113). Increased LHRH density (3000~15000molecules/particle) enhanced targeting while maintaining >60% metabolic activity at therapeutic concentrations (12~30μg/mL), establishing a novel theranostic strategy (113).

Functionalized AuNPs support ovarian cancer-specific detection via fluorescence lifetime imaging (114), dark-field (115), and confocal Raman microscopy (116). SERS imaging further enables chemoresistance prediction and survival outcome assessment (117).

### 3.2 Treatment

#### 3.2 .1. AuNPs-based drug delivery systems

AuNPs enable targeted ovarian cancer drug delivery, enhancing dispersion or internalization of agents like linalool, cetuximab, paclitaxel, let-7a miRNA, and nidocarcinoma-derived factor(**Table 6**). DOX-AuNPs show superior tumor growth inhibition versus free doxorubicin while overcoming payload leakage limitations (118). Engineered for MICU1-targeting siRNA delivery, they achieve >85% silencing efficiency via lysosome evasion, addressing key shortcomings of conventional carriers (119).

**Table 6:** Design of AuNPs for the treatment of ovarian cancer (drug delivery).

No	Particle size	Surface functionalization of AuNPs	Drug	Highlights of Study	Animal model	Toxic	Reference
1	~10nm	Covalent bonding	DTX	Plasmonic photothermal therapy (PPTT) enables pH-triggered controlled release of docetaxel by disrupting halloysite nanotube (HNT) interlayer hydrogen bonds via Au nanoparticle-mediated photothermal effects	–	The MTT results indicate low toxicity in 3T3	(115)
2	~13nm	Thiolation; Covalent bonding	CALNN;Linalool	GSH-capped linalool delivery; CALNN peptide conjugation; Caspase-8/p53 activation & NF-κB inhibition	–	Considered	(120)
3	~40nm	Thiolation	NDC - 1;NDC - 2	Thiol-Au conjugated naproxen derivatives; Simple high-loading platform; 5× lower IC_50_ vs free drugs	–	Considered	(121)
4	~13nm	Thiolation	DOX	DNA-hybridized Dox loading; pH-responsive release; 2.5× greater *in vivo* suppression vs free Dox	BALB/c nude mice	Considered	(118)
5	~20nm	–	auroliposome	AuNP-embedded siRNA liposomes; Caveolae-mediated uptake enhancement; PDX model efficacy	–	No toxicity	(119)
6	12 ± 2 nm	Thiolation	miR - 145	FSH receptor-targeted delivery via FSH33 peptide	–	Considered	(122)
7	~30nm	Electrostatic adsorption; Covalent bonding; Thiolation	PTX	Multimodal imaging (PA/FL/CT); pH-responsive PTX release; Photothermal-enhanced delivery	mice	Considered	(123)
8	~10nm	–	DTX	HNT/AuNP/SORT antibody integration; PPTT/pH-triggered release (44 °C activation); Selective cancer killing	–	The MTT results indicate low toxicity in 3T3	(115)
9	10~15nm	–	TXT	Ca(OH)2 tumor-penetration pretreatment; Magnetic/PPTT/CT multifunctional system; 89% growth inhibition	mice	The MTT results indicate low toxicity in 3T3	(28)
10	60 ± 5nm*30 ± 3nm	Thiolation; Covalent bonding	CSA-131	Peanut-shaped AuNP delivery of ceragenin; Systemic administration potential	BALB/c nude mice	No toxicity	(124)
11	~19nm	Thiolation; Covalent bonding	TfQ	Theaflavin-mediated synthesis/stabilization; Low hemolytic nanocomposite	–	No toxicity	(125)
12	~10nm	Thiolation	TZ; miR-200c	First integration of miR-200c with AuNPs-TZ nanocomplexes demonstrated.	–	Considered	(126)
13	~20nm	Thiolation	DOX	QbD-guided functionalization yielded pH/NIR-responsive AuNPs-L-Dox nanoplatform for selective tumor drug release with reduced systemic toxicity	–	Low toxicity	s(127)

**Figure 9:**
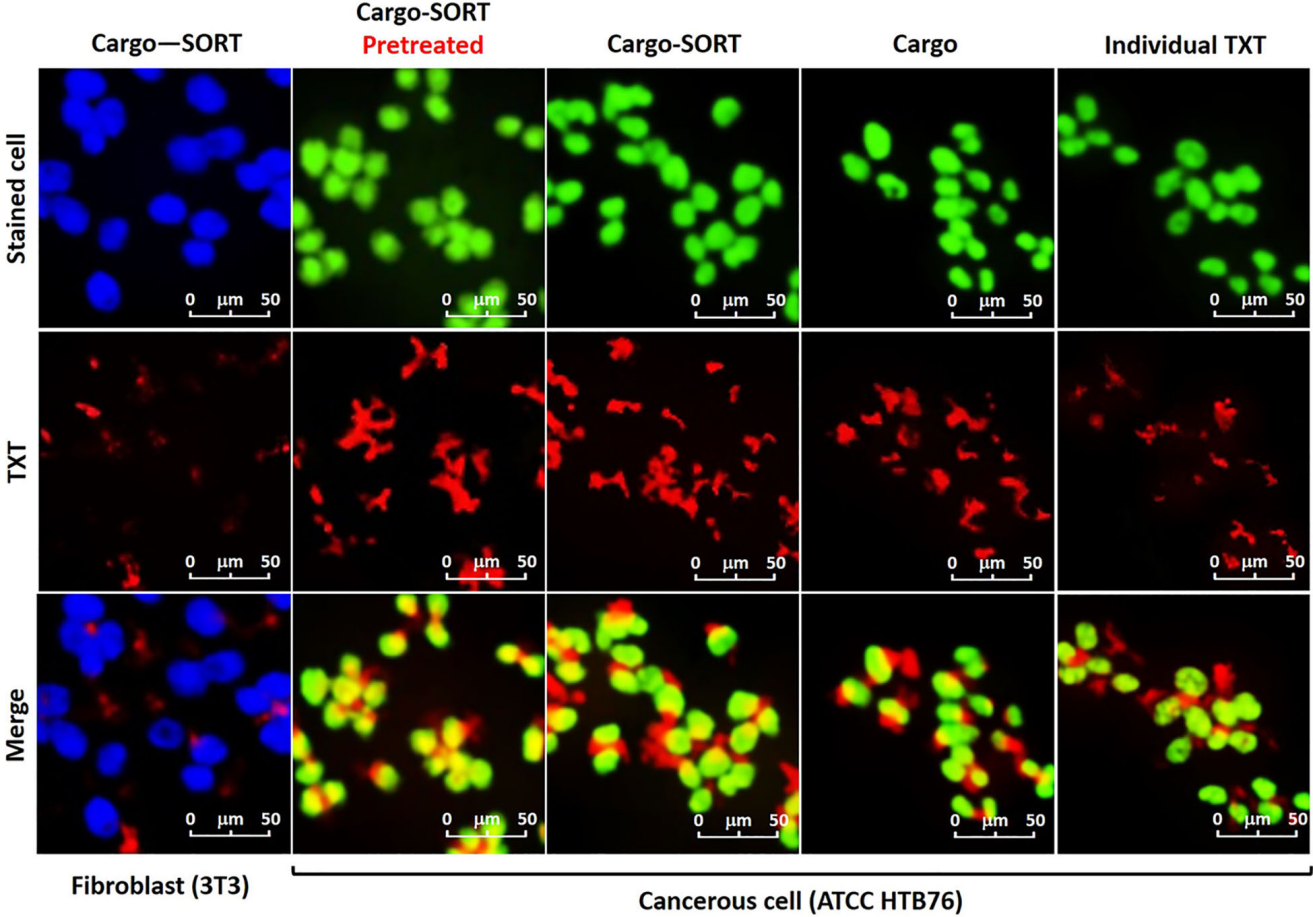
Confocal images of the subjected TXT@Fe3O4/PVA/Au nano-therapeutic to the stained cells. Green: HTB76 cancerous, and blue: NIH 3T3 fibroblast cells (106 DFU), in the presence of the individual TXT, TXT@Fe3O4/PVA/Au (Cargo), and TXT@Fe3O4/PVA/Au-SORT particles (Cargo-SORT) particles. Cell staining was performed using crystal violet, and incubation was carried out at 37 °C with 95% humidity for 2 hours. Pretreatment was done with Ca(OH)2@Fe3O4/PVA/Au-SORT particles in the same dosage with the TXT-containing therapeutic (10μg/mL) (28).

Antibody or peptide-conjugated AuNPs enable precise ovarian cancer targeting (128). Edison and team engineered FSH33-AuNPs covalently conjugated to tumor-suppressive miR-145 (122). This system protects miR-145 from degradation, mediates selective cancer cell internalization, and inhibits proliferation, migration, anchorage-independent growth, and VEGF secretion. AuNPs enable co-delivery of multiple therapeutic agents, as demonstrated by Tommaso et al. who engineered miR-200c and trastuzumab (TZ)-loaded nanoparticles that dual-target critical pathways driving SKOV3 cell survival and proliferation *in vitro*, overcoming TZ resistance while potentially improving therapeutic outcomes for HER2-positive ovarian cancer (126). Beyond antibody/peptide targeting, TME and hyperthermia trigger drug release from AuNPs^,123^. Reza et al. engineered antibody-conjugated magneto-gold composites (TXT@Fe_3_O_4_/PVA/Au-SORT) for ovarian cancer (28). Photothermal AuNPs heating dissociates the PVA matrix, achieving precise TXT release (94.4 ± 4.1% over 180 min in TME). Ca(OH)_2_ pretreatment combined with TXT therapy enhanced intratumoral accumulation, inhibited migration, and induced DNA damage, achieving 78.3% tumor suppression with reduced systemic toxicity (**Figure 9**).

Notably, AuNPs morphology critically influences therapeutic outcomes: anisotropic AuNPs@CSA-131 exhibit enhanced cytotoxicity against ovarian carcinoma versus spherical counterparts (124). Furthermore, AuNPs modulate drug presentation *in vivo*, facilitating theaflavin oxidation to cytotoxic quinone derivatives (125). Regarding therapeutic side effects, The triple-modality approach (ultrasound/AuNCs/cisplatin) overcomes cisplatin resistance in resistant ovarian cancer models, suggesting reduced chemotherapy side effects with translational potential (129).

#### 3.2.2 Intrinsic antitumor effects of AuNPs

AuNPs inhibit ovarian carcinoma invasiveness by targeting key oncogenic pathways: impeding MAPK signaling, suppressing EMT-associated proteins, and disrupting the IGFBP2/mTOR/PTEN autoregulatory axis, downregulating IGFBP2, suppressing PI3K/AKT/mTOR activation, and reactivating PTEN (130, 131). Current understanding posits that AuNPs disrupt multicellular TME communication (cancer cells, cancer-associated fibroblasts, endothelial cells), downregulating pro-tumorigenic cytokines and growth factors (132, 133). Specifically, they reduce CC-secreted fibroblast-activating proteins (TGF-β1, PDGF, uPA, TSP1) and inhibit tumor angiogenesis by blocking VEGF-VEGFR2 signalling (133, 134). This positions AuNPs as key tools for elucidating and disrupting pro-tumorigenic crosstalk. AuNPs synchronize disulfidptosis and ferroptosis in ovarian cancer by modulating the SLC7A11/GSH/GPX4 axis (135). The composite system exploits AuNPs’ glucose oxidase-like activity and Ap-mediated GLUT1 downregulation to induce metabolic crisis (135). Glucose deprivation limits NADPH replenishment, disrupting cystine/cysteine conversion and resolving the disulfidptosis-ferroptosis execution paradox. Concurrently, iron-based components deliver Fe²^+^ while AuNPs-catalyzed glucose oxidation self-supplies H_2_O_2_, amplifying Fenton reactions and ferroptotic death (135).

Beyond influencing signalling pathways, AuNPs enhance nuclear rigidity via perinuclear laminA/C overexpression, impeding cancer cell migration (136). Concurrently, they induce ROS-mediated apoptosis/autophagy, trapping cells in G0/G1 phase (137). Anisotropic AuNPs exert enhanced anti-migratory effects versus spherical counterparts (138). Analogous to cervical cancer applications, morphological engineering of AuNPs enhances their cytotoxic efficacy against ovarian cancer cells. Irfan et al. attribute this phenomenon to elongated-branched antibody-functionalized AuNPs effectively evading serum protein corona entrapment, thereby facilitating optimal aptamer binding to HER2 receptors on cancer cell surfaces (139). This mechanism induces significant cytotoxicity in HER2-overexpressing SKOV3 cells through targeted apoptosis initiation.

#### 3.2.3 High-efficiency photothermal tumor ablation utilizing AuNPs

AuNPs enable synergistic chemo-photothermal therapy for ovarian cancer (127, 140). Yiting et al. engineered a genetically fused HSA nanocarrier (RHMH18@AuD) self-assembling via histidine hydrophobicity to encapsulate DTX while forming ultrasmall AuNPs through biomimetic mineralization (141). This 80-nm platform prevents HSA denaturation and reduces inorganic nanoparticle toxicity. MMP-2 cleavage at tumors releases RGD-HSA@Au (mediating photothermia) and His@DTX micelles, with acidic TME-triggered DTX release. The system demonstrated targeted cellular uptake, significant tumor suppression, and 100% survival at 70 days versus complete mortality in monotherapy groups by day 62, establishing a high-efficacy, low-toxicity therapeutic strategy.

Diverse AuNPs composites serve as photothermal agents for ovarian cancer PTT. rGO-AuNPs-PEG exhibits strong SERS signals, NIR-II PA signals, and high photothermal efficiency in tumours under 1061 nm laser irradiation (142). Similarly, silica nanocapsules containing aggregated AuNPs yolk-shell structures (aAuYS) demonstrate enhanced photothermal effects with 808 nm laser exposure (143). Curcumin-incorporated gold nanoshells (Cur-AuNShs) show efficient photothermal conversion with potential for selective cancer targeting and treatment. Additionally, AuNPs morphology influences PTT efficiency (144). For instance, dumbbell-shaped Au-Fe_3_O_4_ elevate thermal conversion efficacy (145).

To overcome resolution limitations in image-guided PTT, Annan et al. developed ultra-small GnRHR-targeted AuNDs (Au-GRHa, 3.2nm) (146). Prepared via electrochemical displacement and ligand conjugation, these nanoconstructs enable dual-modal fluorescence/CT imaging with superior CT contrast (attenuation coefficient: 5.153cm²/g) and renal clearance. GnRHa targeting boosted SKOV3 cellular uptake by 76% versus non-targeted counterparts. Under 808 nm irradiation, localized temperatures reached 50 °C within 5 min, inducing apoptosis via membrane disruption and protein denaturation. *In vivo* peak tumor accumulation occurred at 2 h, with subsequent PTT significantly suppressing tumor growth without hemolysis or toxicity, establishing a precise image-guided therapeutic platform.

PDT and PTT act synergistically against ovarian cancer. A multifunctional nanomicrogel (Au@MSN–Ter/THPP@CM@GelMA/CAT) demonstrates concurrent photodynamic efficacy (650nm) and photothermal ablation (980nm) (147).

#### 3.2.4 Radiosensitization effects of AuNPs in radiotherapy

**Figure 10:**
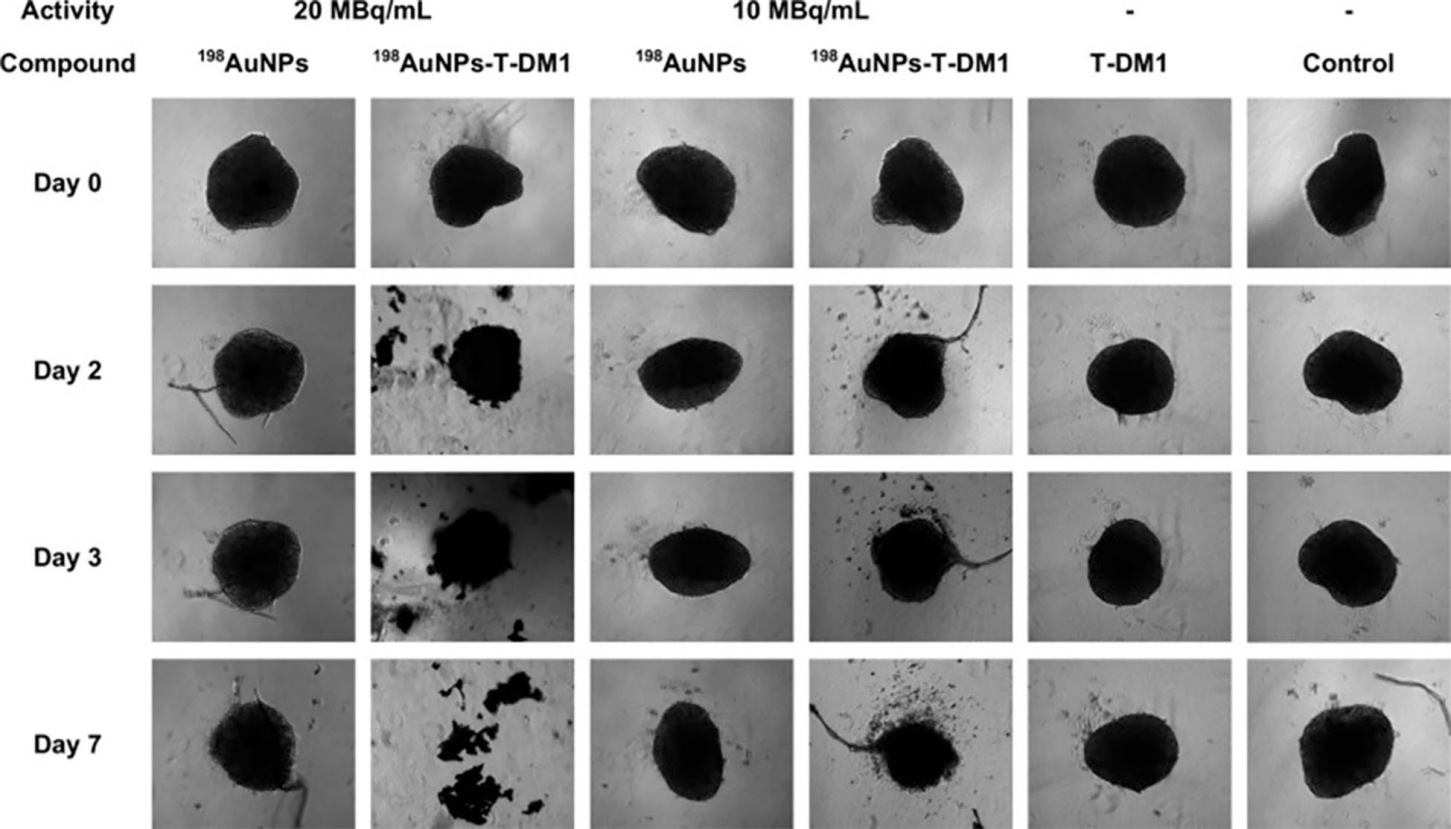
Microscopic images of the measured control and compound-treated SKOV-3 spheroids (149).

AuNPs exhibit radiosensitizing effects, exemplified by thioglucose-bound nanoparticles (Glu-GNPs) enhancing ovarian cancer radiotherapy (148). The GO-SPIO-Au nanoflower platform integrates graphene oxide (NIR-PTT), AuNPs (radiosensitization), and superparamagnetic iron oxide (MRI) for image-guided therapy (112). In murine models, combined PTT/RT yielded 1.85× and 1.44× higher efficacy than PTT or RT alone, respectively. Kinga et al. developed a novel cancer therapy combining antibody-drug conjugates (ADCs) with β-emitting ¹^98^AuNPs conjugated to trastuzumab emtansine (T-DM1), demonstrating specific HER2 affinity and synergistic efficacy against HER2-overexpressing cancers at low T-DM1 doses (0.015~0.124μg/mL) with 10–20 MBq/mL radiation (149). Continuous 7-day treatment (20 MBq/mL+0.031μg/mL T-DM1) disrupted 3D tumor spheroids, suggesting potential for HER2-positive breast/ovarian cancer treatment despite preferential suitability of inorganic nanoradiopharmaceuticals for localized delivery (**Figure 10**) (149).

## 4 Integrated diagnostic and therapeutic platform

AuNPs serve as pivotal components in multimodal theranostic platforms owing to their distinctive physicochemical properties, enabling visualized precision therapy of pathological lesions. Diversified therapeutic strategies demonstrate that targeted accumulation of AuNPs at disease sites generates synergistic effects, with imaging guidance being critical for maximizing diagnostic-therapeutic efficacy. Current research has developed functionalized AuNPs-based visualization approaches for gynecological malignancies. Zhang’s team overcame single-modality imaging limitations by establishing a PAI/CT/MRI multimodal system centered on functionalized AuNPs, effectively addressing the low X-ray attenuation coefficient inherent to inorganic materials (67). At 200 µg/mL, Au-UCNPs-DSPE-PEG precisely delineated cervical cancer location, dimensions and morphological characteristics in murine models, while their photothermal conversion capability simultaneously enabled photoacoustic imaging-guided combination photothermal and photodynamic therapy (67). Beyond physical modalities, AuNPs function as chemotherapeutic carriers. Taheri et al. employed CT to monitor TXT@AuNPs distribution for efficacy assessment, yet single-modality CT proved inadequate for tracking drug release kinetics (28). Wang’s team addressed this through multimodal imaging (PAI/FL/CT) for real-time surveillance of drug-loaded AuNPs. Paclitaxel release induced fluorescence signal fluctuations due to AuNP surface restructuring while accelerating nanoparticle metabolic clearance, consequently reducing photoacoustic intensity in lesions (123). For quantitative release monitoring, Yim’s team innovatively leveraged AuNPs’ low X-ray attenuation property (66). Electrostatic adsorption-triggered aggregation of radioiodinated AuNPs, occurring through opposite surface charges after DOX release, significantly enhanced lesion signals on PET-CT imaging (66). These aggregates maintained prolonged high-signal states due to extended half-life, enabling sustained dynamic observation.

## 5 Challenges and limitations

Despite being a promising nanomaterial, AuNPs must overcome several significant barriers prior to broad clinical adoption for diagnosing and treating gynecological malignancies.

The safety profile of AuNPs represents a modifiable property, critically dependent on factors such as particle size, synthesis method, exposure route, dosage duration, and the specific biological milieu (150, 151). In numerous studies cited above, AuNPs are often assumed to be chemically inert and stable materials, particularly when PEG-modified, exhibiting negligible toxicity at certain doses. Merely 28 investigations to date have employed MTT assays and related techniques to evaluate the cytotoxicity of novel functionalized AuNPs toward normal cells or animal models. In the clinical context of managing gynecological malignancies, chemotherapy is typically a protracted process (152). Repeated administration of functionalized AuNPs during such long-term treatment carries a significant risk of inducing antibodies against the nanoparticle surface characteristics. This immunogenic response could potentially compromise the targeting efficacy of AuNPs and disrupt normal immune function. Furthermore, diverse AuNPs synthesis and functionalization strategies can leave toxic chemical residues on the particle surfaces. These modifications also alter the chemical properties and size of the AuNPs, potentially hindering renal clearance and leading to progressive bioaccumulation. Additionally, compared to free drugs, administering chemotherapeutic nanoparticles during ovulation increases ovarian toxicity and reduces fertility (153). Therefore, the menstrual cycle warrants consideration in the design and implementation of AuNPs therapies for female patients. These concerns underscore the necessity for further comprehensive evaluation of AuNPs systemic safety in humans and detailed investigation into nanoparticle pharmacokinetics to fully assess their absorption, distribution, metabolism, and excretion processes.

Addressing AuNP safety challenges requires systematic pharmacokinetic studies (absorption, distribution, metabolism, excretion) in animal models to define critical thresholds for nanoparticle-induced irreversible organ damage, enabling establishment of dimensionally-, morphologically-, and synthesis-method-dependent safety dosage windows across varied administration regimens. Green synthesis strategies utilizing novel catalysts demonstrate promising toxicological safety profiles, potentially representing key advancement pathways (75, 76). Multifunctional AuNPs may shorten chemotherapy cycles while combined PTT and PDT therapies could circumvent antibody responses from chronic treatment (127, 141). Nevertheless, large-scale animal validation remains indispensable; current maximum reported cohort sizes of 28 subjects prove insufficient, particularly given physiological disparities between rodent and human systems, necessitating expansion to rabbit and non-human primate models. These imperatives collectively emphasize comprehensive assessment of systemic AuNP safety in humans and rigorous pharmacokinetic investigation.

The diagnostic and therapeutic efficacy of AuNPs requires further validation. The majority of studies demonstrating potential benefits are confined to cell lines or small animal models, overlooking the substantial complexity of human physiology. In cervical cancer diagnostics, merely 15 of 34 peer-reviewed investigations disclosed clinical sample accuracy (n=9) or spiked serum analyte recovery (n=6), with two HPV detection reports achieving >95% accuracy in cohorts exceeding 100 specimens. Regarding therapeutic applications, only 2 of 15 cervical cancer publications documented AuNP efficacy in murine models. Similarly, among 28 ovarian cancer diagnostic analyses, 18 provided clinical validation data (n=4) or serum recovery metrics (n=14), though clinical specimens numbered ≤10 per analysis. Whereas 21 of 28 therapeutic investigations asserted significant antitumor outcomes, merely 9 confirmed efficacy in animal models with quantification parameters undisclosed. Humans are continuously exposed throughout life to diverse natural and anthropogenic nanoparticles. Such environmental nanoparticle contamination constitutes a significant exogenous interference factor, potentially impeding the function of administered AuNPs. For instance, titanium or iron oxide nanoparticles can inhibit cancer cell uptake of AuNPs (154). Beyond these exogenous factors, endogenous human variables also critically influence AuNPs performance. Evidence indicates that elevated cholesterol levels and specific lipid ratios disrupt the delivery capacity of DOX-AuNPs systems (155). Even in Phase III clinical trials, AuNPs-based drug delivery systems demonstrated suboptimal recognition efficiency for ovarian cancer, with the majority of intratumoral nanoparticles becoming either trapped within the extracellular matrix or sequestered by perivascular tumor-associated macrophages (156). Compounding these issues, many studies report human validation based on single-digit sample cohorts, lacking comparison with healthy individuals or non-gynecological cancer patients, and frequently omit detailed accuracy data (**Supplementary Material 1**).

Subsequent investigations must validate AuNPs’ true diagnostic-therapeutic efficacy through large-scale animal models and clinical trials, with priority assessment of their resistance to complex biological interferences including protein corona formation and lipid adsorption. Although functionalized AuNPs demonstrate anti-interference capabilities in select studies, the disparity between simulated laboratory conditions and physiological environments necessitates rigorous *in vivo* verification. Novel non-spherical geometries such as high-aspect-ratio nanostars effectively circumvent protein corona shielding while enhancing tumor targeting precision (139). Notably, nanoparticles within the 10~20 nm size range exhibit optimal performance, yet synthesis-dependent variations in AuNP dimensions/morphologies demand standardized evaluation frameworks to enable cross-study comparability and collaborative advancement. Furthermore, addressing prevalent data limitations stemming from insufficient clinical samples requires establishing multicenter validation frameworks. AuNPs’ therapeutic potential should transcend conventional drug delivery roles toward multimodal theranostic platforms, exemplified by triple-modality regimens integrating PTT, controlled chemotherapeutic release, and radiosensitization, with concurrent treatment monitoring via PAI and PET-CT. While four diagnostic investigations have incorporated machine learning for enhanced SERS-based high-throughput chip detection, deep learning applications in medical image interpretation remain unexplored. Integrating big data analytics with mobile health technologies could establish intelligent diagnostic networks to reduce misinterpretation risks.

Finally, the cost implications of AuNPs systems demand serious consideration. In resource-limited developing nations, economic constraints remain pivotal in restricting large-scale disease screening initiatives. Most current studies fail to address the cost structure of AuNPs-based diagnostic platforms, with only a handful reporting screening expenses or reusability metrics (157). A predominant focus on novel materials and intricate architectures, particularly acute within the domain of AuNPs-designed electrochemical sensors, often overshadows the underlying premise of screening: low cost and high accessibility. Furthermore, AuNPs synthesis methodologies themselves represent significant cost determinants, compounded by concerns regarding environmental impact and suboptimal production efficiency (151). These factors establish cost as a paramount consideration for the clinical translation of AuNPs technologies.

The convergence of artificial intelligence and low-cost smartphones offers a significant pathway to reduce expenditures associated with AuNP-based diagnostic systems, effectively lowering human resource requirements, time costs, and sample transport losses. Implementing a three-tier diagnostic network comprising colorimetric detection units, subject mobile client devices, and hospital data centres substantially enhances population screening efficiency. A core advantage of this system lies in the capacity for AI algorithms to perform localized processing directly on smartphones, enabling preliminary screening and interpretation of test results; only data indicating anomalies require transmission to the hospital data centre for verification, thereby markedly alleviating the healthcare burden in resource-limited settings. This tiered network fundamentally transforms the traditional hierarchical “hospital-centric–healthcare worker–subject” information delivery model. By empowering subjects with autonomous testing capabilities, it shifts the paradigm from passive information reception to proactive health management, significantly improving participant engagement and adherence. From a technological development perspective, research efforts should recalibrate their focus regarding AuNPs: prioritizing material design optimization that establishes an optimal cost-accuracy balance over the pursuit of increasingly complex material combinations; directing energy towards developing scalable, low-power manufacturing processes; and advancing clinical integration through modular designs that reduce the overall system cost.

**Figure 11:**
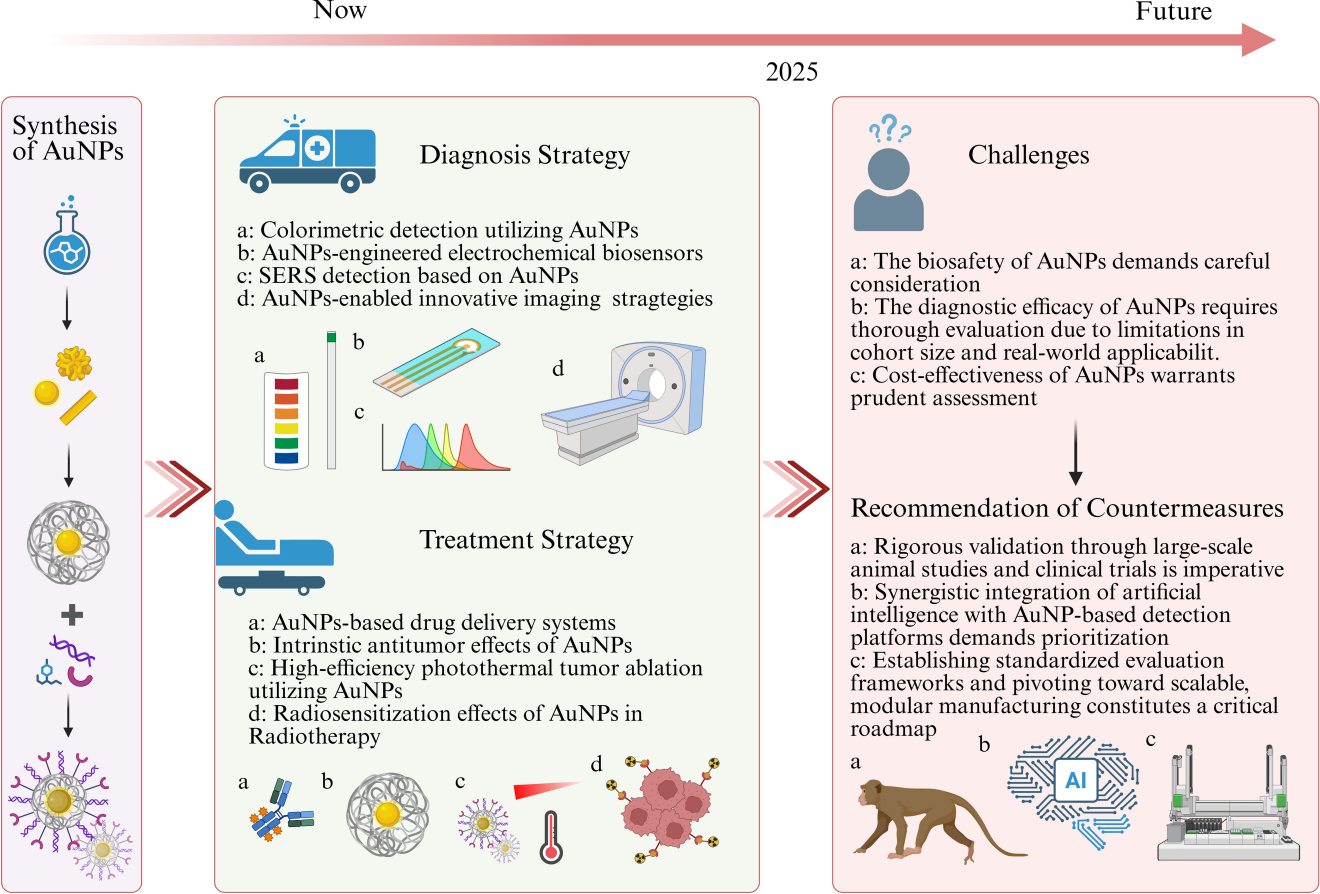
Current diagnostic and therapeutic approaches utilizing AuNPs in cervical and ovarian cancers, and associated challenges and future prospects.

Notably, clinical trials of AuNPs in gynaecological malignancies remain limited. However, recent human studies across non-gynaecological cancers, spanning breast cancer, colorectal carcinoma and cutaneous disorders, demonstrate expanding clinical evaluation (158–162). These advances confirm that current implementation challenges are addressable and reveal diagnostic and therapeutic benefits warranting further translation in gynaecological oncology. Collectively, AuNPs systems exhibit significant potential for enhancing diagnostic accuracy, improving patient quality of life, and optimizing clinical prognoses. Looking forward, their unique physicochemical properties position AuNPs as transformative agents in next-generation gynecologic oncology, enabling minimally invasive theranostics, real-time disease monitoring, and personalized treatment regimens. Continued advancements in nanomaterial engineering, refined targeting methodologies, comprehensive safety evaluations, and integration of AI further solidify AuNPs platforms to assume an increasingly critical and expansive role in future integrated theranostic frameworks (**Figure 11**).

## 6 Conclusion

Clinical management of gynecological malignancies faces significant challenges, including difficulties in early detection, high therapeutic resistance, substantial risks of residual disease post-surgery, and considerable toxicity from conventional radiotherapy and chemotherapy. These critical limitations demand innovative technological solutions for precision diagnostics and therapeutics. Recent advances in nanotechnology provide transformative momentum for gynecologic oncology, with AuNPs offering particularly promising strategies due to their tunable dimensions, morphological versatility, customizable surface functionalization, and unique optical properties. AuNPs serve as highly sensitive contrast agents that enhance detection rates for early-stage lesions and micrometastases. Functionalization with antibodies, peptides, or aptamers enables precise targeting of therapeutic payloads to disease sites and facilitates ultrasensitive detection of trace biomarkers in liquid biopsies. Furthermore, their exceptional photothermal conversion efficiency and photochemical capabilities permit concurrent targeted chemotherapy with spatially precise photothermal and photodynamic therapy at tumor sites. This integrated theranostic approach positions AuNPs-based systems to drive a paradigm shift from isolated interventions toward closed-loop precision management in gynecologic oncology. Nevertheless, further validation remains imperative to address clinical translation barriers and long-term safety profiles.

## Glossary

FDA: US Food and Drug Administration
PEG: Polyethylene glycol
OCT: Optical coherence tomography
VIA: Visual inspection with acetic acid
LOD: Limit of detection
LFA: Lateral flow assay
PDA: Polydopamine
PEBA: Photoelectrochemical biochip array
MLNPs: Multilayered nanoparticles
WS_2_: Tungsten disulfide
MoS_2_: Molybdenum disulfide
ND: Neutral density filter
4-MBA: 4-Mercaptobenzoic acid
DTNB: 5,5′-Dithiobis-(2-nitrobenzoic acid)
4,4′-DP: 4,4′-Dipyridyl
RGD: Arginylglycylaspartic acid peptide
NLS: Nuclear localization signal
TAP: Tris(2-aminophenol)
PHEN: 1,10-Phenanthroline
3-MPA: 3-Mercaptopropionic acid
TLR7/8: Toll-like receptors 7/8
MPA: 3-Mercaptopropionic acid
MPS: 3-Mercaptopropyltrimethoxysilane
PVP: Polyvinylpyrrolidone
ECM: Extracellular matrix
RNS: Reactive nitrogen species
MB: Methylene blue
Pp-IX: Protoporphyrin IX
AuNRs: Gold nanorods
DEF: Dose enhancement factor
FFPE: Formalin-fixed paraffin-embedded
AGR2: Anterior gradient 2
Mg/Fe LDH: Mg/Fe-layered double hydroxide
PAMAM: Polyamidoamine
MXene: Transition metal carbide/nitride
MOF: Metal-organic framework
GQD: Graphene quantum dot
DLS: Dynamic light scattering
TIMO^+^F-KB@AuNPs: Thiolated Ionic Metal-Organic Framework-KB@AuNPs
PB: Prussian blue
CoFeB: Cobalt iron boride
CDDP: Cisplatin
sEV: Small extracellular vesicles
VOCs: Volatile organic compounds
HYNIC: Hydrazinonicotinamide
GO/SPIO/AuNP: Graphene oxide/superparamagnetic iron oxide/gold nanoparticle
ICP-MS: Inductively coupled plasma mass spectrometry
FLIM: Fluorescence lifetime imaging microscopy
SCRM: Scanning confocal Raman microscopy
FSH33: Follicle-stimulating hormone peptide fragment
VEGF: Vascular endothelial growth factor
PVA: Poly
AuNCs: Gold nanoclusters
PDGF: Platelet-Derived Growth Factor
uPA: Urokinase-Type Plasminogen Activator
TSP1: Thrombospondin-1
Fe-Ap: Iron-Apigenin
AuNSs: Gold nanostructures
HSA: Human Serum Albumin
MMP-2: Matrix Metalloproteinase-2
rGO: Reduced Graphene Oxide
aAuYS: Aggregated Gold Yolk-Shell
Cur-AuNShs: Curcumin-Incorporated Gold Nanoshells
GnRHR: Gonadotropin-releasing Hormone Receptor
AuNDs: Gold nanodots
EDC: 1-Ethyl-3-(3-dimethylaminopropyl)carbodiimide
NHS: N-Hydroxysuccinimide
Au@MSN: Gold-coated Mesoporous Silica Nanoparticles
Ter: Terthiophene
THPP: Tetrakis(4-hydroxyphenyl)porphyrin
GelMA: Gelatin Methacryloyl
sNPS: Single-Nanoparticle Sensing System
MutS: DNA Mismatch Repair Protein
EpCAM: Epithelial cell adhesion molecule
COF: Covalent Organic Framework
Apt: Aptamer
LDI-MS: Laser Desorption/Ionization Mass Spectrometry
EMS: Endometriosis
EphB4: Ephrin type-B receptor 4
IDEs: Interdigitated electrodes
CDI: 1,1′-Carbonyldiimidazole
MWCNT: Multi-walled carbon nanotubes
APTES: 3-Aminopropyl)triethoxysilane
SA-HRP: Streptavidin-Horseradish Peroxidase
GCE: Glassy carbon electrode
ZnO NRs: Zinc Oxide Nanorods
ERGO: Electrochemically Reduced Graphene Oxide
SPCE: Screen-Printed Carbon Electrode
FTO: Fluorine-Doped Tin Oxide
TDN: Tetrahedral DNA Nanostructure
TMSD: Toehold-Mediated Strand Displacement
ZIF: Zeolitic Imidazolate Framework
PTH/PTB: Polythiophene
DSPCE: Double-Screen Printed Carbon Electrode
DES: Deep Eutectic Solvent
CuCo-ONSs: Copper-Cobalt Oxide Nanosheets
